# Morphology-Dependent Electrochemical Performance of Zinc Hexacyanoferrate Cathode for Zinc-Ion Battery

**DOI:** 10.1038/srep18263

**Published:** 2015-12-16

**Authors:** Leyuan Zhang, Liang Chen, Xufeng Zhou, Zhaoping Liu

**Affiliations:** 1 Ningbo Institute of Materials Technology and Engineering, Chinese Academy of Sciences, Zhejiang 315201, P.R. China

## Abstract

Zinc hexacyanoferrate (ZnHCF) which is a dimorphic (cubic or rhombohedral) Prussian blue analogue and can be intercalated by both monovalent and divalent ions,is a promising cathode material for rechargeable aqueous metal-ion batteries.In this paper, a simple co-precipitation method is developed to tune the particle morphology of ZnHCF by adjusting the dropping speed at room temperature. Three polyhedral ZnHCF particles, with cubooctahedral, truncated octahedral or octahedral shapes, are obtained at room temperature. Structural transformation from cubic phase of as-prepared ZnHCF to rhombohedral phase is observed by further dehydration of the sample at 70 °C, whereas the dehydrated ZnHCF crystals still hold the identical polyhedral shape as that of the cubic phase particles. Then the influence of shape and facets on electrochemical performance is studied for polyhedral ZnHCF with rhombohedral structure (RZnHCF). RZnHCF sample with cubooctahedral shape possesses the best rate capability and cyclic stability comparing with RZnHCF particles having truncated octahedral or octahedral shapes. Furthermore, the structure of cuboctahedron RZnHCF particles during electrochemical cycling has been monitored with *ex situ* X-ray diffraction to demonstrate the reversible zinc-ion intercalation mechanism.

Due to the advantages of low cost, environmental benignness and high ionic conductivity, rechargeable aqueous metal-ion batteries (RAMB) have a giant application potential in large-scale energy storage. “Rocking-chair” RAMB based on monovalent ions (Li^+^, Na^+^ or K^+^) have been investigated extensively, but most of them show the shortage of low energy density due to the low operating voltage(<1.5 V)^1^,^2^,^3^,^4^,^5^,^6^,^7^,^8^,^9^,^10^,^11^,^12^,^13^,^14^,^15^. Multivalent metal-ion (Mg^2+^, Zn^2+^, Al^3+^, etc.) RAMB are attracting more and more attention because of their potentially higher energy. But only few systems regarding multivalent ions intercalation chemistry have been reported^16^,^17^,^18^, ascribing to the limits of electrode materials.

In recent years, Prussian blue analogues (PBAs) are widely studied in the energy storage systems as the electrode materials^19^,^20^,^21^,^22^,^23^. Among PBAs, metal hexacyanoferrates(MeHCFs) with open-framework structures can not only be inserted by monovalent alkaline cations^24^ such as Li^+^, Na^+^ or K^+^, but can also behave as hosts for divalent or trivalent cations^25^,^26^,^27^ including Mg^2+^ and Al^3+^. Among the reported RAMB with MeHCFs as electrode materials^14^,^26^,^28^,^29^, most MeHCFs such as CuHCF, NiHCF or CoCuHCF have cubic structure, and the voltages of the batteries are generally lower than 1.5 V due to the relatively high potential of applicable anode materials. Comparing with most reported anode materials in RAMB, metal zinc shows apparently lower potential of –0.78 V vs. standard hydrogen electrode potential. Moreover, the electrical deposition/dissolution of zinc is highly reversible. Therefore, it provides a viable strategy to achieve high operating voltage in RAMB based on zinc ions. But the lack of cathode materials allowing reversible insertion/extraction of divalent Zn^2+^ in aqueous electrolytes greatly hinders the development of aqueous zinc-ion batteries. To the best of our knowledge, among the known cathode materials only an α-MnO_2_ cathode material can permit the reversible intercalation of divalent Zn^2+^ in aqueous electrolytes^16^. Fortunately the structural diversity of MeHCFs makes it possible to design new high-performance divalent metal-ion RAMB, including aqueous zinc-ion battery. Recently, we reported the use of ZnHCF as a novel cathode material in RAMB based on zinc ions for the first time^30^. Combining with Zn as the anode, the Zn/ZnHCF system can exhibit a high operating voltage of 1.7 V, giving rise to a high energy density of 100 Wh kg^−1^.

Compared to most of MeHCFs with cubic structure, ZnHCFs exhibit the rhombohedral structure in which the ZnN_4_ tetrahedras are linked to FeC_6_ octahedras via CN ligands to form a porous three-dimensional framework with large open sites where metal ions such as Na^+^, K^+^ and Cs^+^ and water molecules are located. Such a framework endows ZnHCFs with the possibility of acting as intercalation hosts for various cations. The structural difference between ZnHCFs and common MeHCFs will undoubtedly lead to different intercalation mechanisms of metal ions. The ions intercalation mechanisms in the cubic MeHCFs have been studied intensively^12^,^28^,^29^. The feasibility of ZnHCFs as intercalation host for Na^+^ has also been demonstrated by Chio and coworkers^23^. However, the ions intercalation mechanism of divalent metal ions, such as zinc ions in ZnHCF remains to be further studied. This is the first study on the intercalation mechanism for divalent Zn^2+^ in ZnHCF. Moreover, the effect of the shape and facets of the ZnHCF particles on their electrochemical performance was also systematically investigated in this work. The morphology and crystal-facet-controlled synthesis of cathode materials have recently attracted considerable attention^31^,^32^,^33^ and a number of shape-controlled PBAs can be synthesized^34^,^35^,^36^,^37^. For example, Guo’s group^38^ found that high-quality Prussian blue crystals in the form of nanocubes showed a better electrochemical performance than low-quality crystals with a granular morphology. By controlling the reaction conditions, uniform ZnHCF particles with a series of regular morphologies were successfully prepared, and their relationship between exposed facets and electrochemical performance was proposed, which will give new insights into the design of high-performance RAMB based on PBAs.

## Results

### Shape-controlled Synthesis

**Figure 1:**
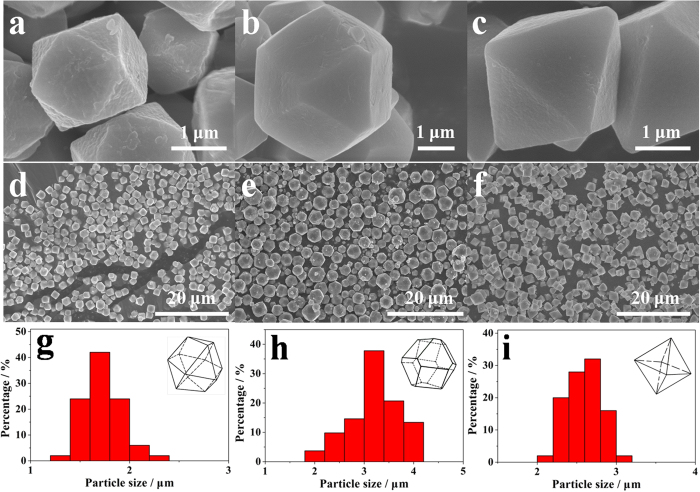
SEM images of (**a,d**) cuboctahedron ZnHCF, (**b,e**) truncated octahedron ZnHCF and (**c,f**) octahedron ZnHCF obtained at room temperature, and their size distribution histograms (*g*) for cuboctahedorn ZnHCF, (**h**) for truncated octahedron ZnHCF and (**i**) for octahedron ZnHCF, respectively) plotted by counting 50 random particles.

In our previous work, the ZnHCF was synthesized by a conventional co-precipitation reaction between 0.1 M ZnSO_4_ and 0.05 M K_3_Fe(CN)_6_ aqueous solutions and irregular particles were obtained (Fig. S1a)^30^. In this work, by reducing the concentrations of aqueous ZnSO_4_ and K_3_Fe(CN)_6_ solutions to 0.01 M and adjusting the dropping speed of the reactant solutions, uniform ZnHCF particles with a series of polyhedral shapes can be prepared at room temperature, as shown in Fig. 1. When the aqueous solution of 0.01M ZnSO_4_ and aqueous solution of 0.01M K_3_Fe(CN)_6_ were poured simultaneously into one beaker, ZnHCF particles with cuboctahedron shape were obtained (Fig. 1a). If the reactant solutions were mixed slowly by using dropping pipettes in approx. 20 minutes, the shape of ZnHCF particles changed to be truncated octahedron (Fig. 1b). If the mixing process of the reactant solutions was further elongated to 2 hours by using peristaltic pump, the shape of ZnHCF crystals became octahedron (Fig. 1c). Figure 1d-f display the overall morphology of shape-controlled ZnHCF particles in a larger domain, which verifies good homogeneity in both shape and size of ZnHCF particles in each sample. The size distribution of ZnHCF particles is statistically plotted in Fig. 1g–i. The particle sizes of all three samples are distributed within a narrow range of 1–5 μm. The mean dimension, however, varies slightly between polyhedral particles with different shapes. The cuboctahedron ZnHCF (named as C-ZnHCF hereafter) particles possess the smallest mean size of ~1.7 μm, and the truncated octahedron ZnHCF (named as T-ZnHCF hereafter) particles have the largest mean size of ~3.2 μm, whereas the average size of octahedron ZnHCF (named as O-ZnHCF hereafter) lies in between (~2.6 μm).

**Figure 2:**
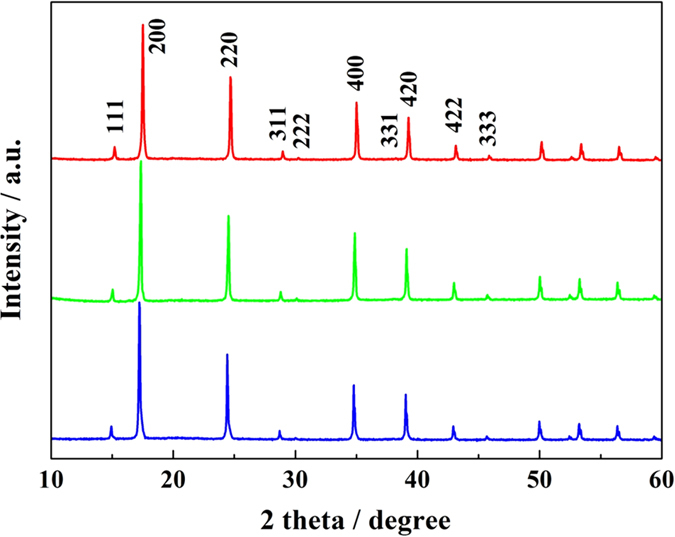
XRD patterns of cubic phase ZnHCF with cubooctahedral (red),truncated octahedral (green) and octahedral (blue) shapes obtained at room temperature.

XRD analysis (Fig. 2) of all three morphology-controlled ZnHCF samples dried at room temperature shows identical diffraction patterns of cubic phase Zn_3_[Fe(CN)_6_]_2_•xH_2_O (PDF#38-0687). So it is easy to deduce that all three polyhedral ZnHCF particles are bound by entirely {100} and/or {111} facets of the fcc crystal symmetry. The C-ZnHCF is bound by {111} and {100} planes, and the ratio between surface area of {111} facets (S111) to that of {100} facets (S100) is estimated to be 0.6. The T-ZnHCF has a higher S111/S100 ratio of ca. 1.2, and the O-ZnHCF particles are almost entirely capped by {111} facets.

**Figure 3:**
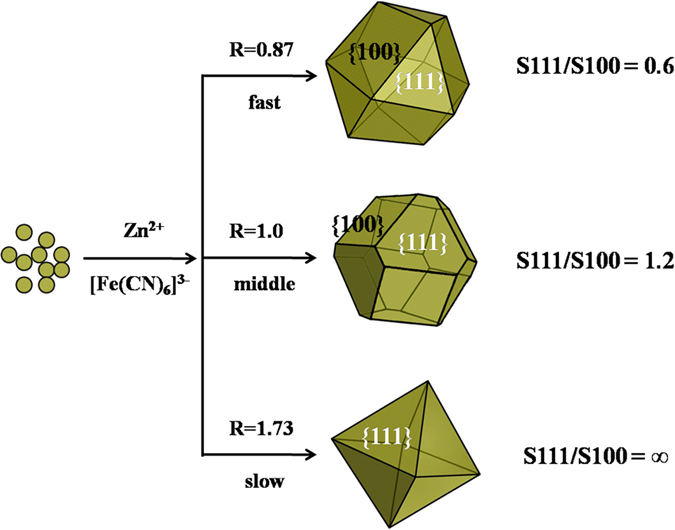
A schematic illustration of nucleation and growth process that determines the shape of ZnHCF particles obtained with different dropping speed of reactants; the right is the ratio of surface area of facets {111} to {100}.

The morphology of ZnHCF crystals is strongly dependent on the concentration of the reactants. The SEM images of ZnHCF particles synthesized at different concentrations are shown in Fig. S2. At either higher or lower concentrations than 0.01 M, irregular particles with no characteristic morphologies were obtained. The concentration of 0.01 M was found to be the optimal condition to obtain uniform ZnHCF particles with regular morphologies. When ZnSO_4_ and K_3_Fe(CN)_6_ aqueous solutions were quickly poured into the beaker, the orange-red precipitate was obtained immediately, indicating fast nucleation. As seen in the SEM image (Fig. S3), the uniform ZnHCF crystals were immediately formed in the solution without the process of agitation and age. What’s more, the dropping speed of reactants which affects the growth of crystals also plays a crucial role in determining the polyhedral shape of theproducts. The formation mechanism of ZnHCF crystals with controlled morphology is illustrated in Fig. 3, which can be attributed to the homogeneous nucleation and growth of the crystals. Wang’s group^39^ has previously observed that the formation of various different shapes of an fcc nanocrystal depended on the ratio (R) of the growth rate along <100> to that along <111>, and geometrical shapes of cubooctahedral nanocrystals as a function of the ratio R were illustrated accordingly. According to Wang’s theory, the dropping speed of the reactants may affect the R values to determine the shape of ZnHCF crystals in this work and the R values of cuboctahedron, truncated octahedron and octahedron ZnHCF should be 0.87, 1.0 and 1.73, respectively.

Above all, we propose in this paper a simple co-precipation method to prepare shape-controlled ZnHCF samples. Although someshape-controlled Prussian blue^40^ and its analogues^34^,^35^,^36^,^37^ such as Mn_3_[Co(CN)_6_]_2_, Co_3_[Co(CN)_6_]_2_ and Zn_3_[Co(CN)_6_]_2_ have been previously observed, surfactants or hydrothermal process were required in those reports. In our work, ZnHCF particles with different regular morphologies could be obtained by controlling the concentrations and adjusting the dropping speed of reactants in the absence of surfactants at ambient conditions. Furthermore, this simple reaction procedure can also be applied as a general method to synthesize other PBAs with regular shapes, such as manganese hexcyanoferrate (MnHCF), cobalt hexacyanoferrate (CoHCF) and silver hexacyanofarrate (AgHCF), whose SEM images and XRD diffraction patterns are shown in Fig. S4.

### Structure Transformation

**Figure 4:**
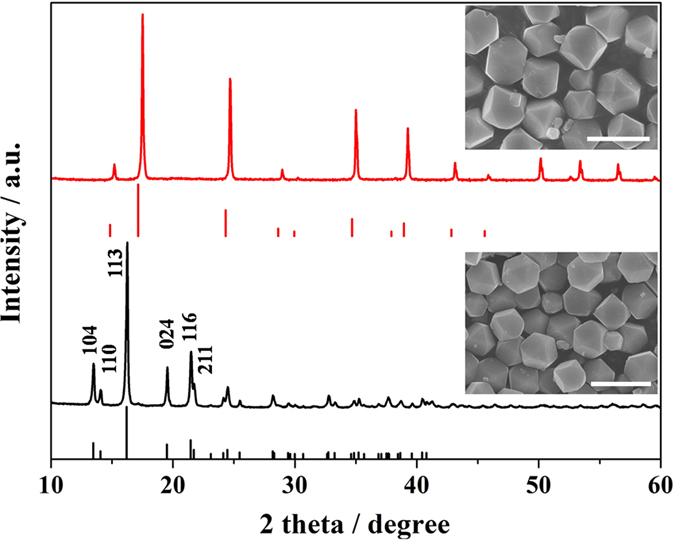
The XRD patterns of cuboctahedron ZnHCF dried at room temperature with cubic structure(red) and the one (black) further dried at 70 °C with rhombohedral structure. Insets are the corresponding SEM images of two samples (scar bar 3 μm).

As mentioned in some works^41^,^42^, Zn_3_[Fe(CN)_6_]_2_ •xH_2_O with cubic structure is not stable. The water molecules are very easy to escape by heat treatment, transforming its structure to be rhombohedral, which also occurred in our research. Taking C-ZnHCF as an example, when it was further dried at 70 °C for 12 hours, the sample was dehydrated and transformed to be rhombohedral structure (Zn_3_[Fe(CN)_6_]_2_, PDF#38-0688), as reflected by the XRD pattern shown in Fig. 4. However, the cubooctahedral shape of C-ZnHCF is well preserved after the structural transformation. The same phenomenon was also observed in T-ZnHCF and O-ZnHCF when heated at 70 °C for 12 hours, as shown in Fig. S5. The chemical formula of rhombohedral phase ZnHCF (RZnHCF) with cuboctahedron, truncated octahedron and octahedron shapes was found to be K_0.07_Zn[Fe(CN)_6_]_0.69_, K_0.08_Zn[Fe(CN)_6_]_0.67_ and K_0.07_Zn[Fe(CN)_6_]_0.68_ respectively, by inductively coupled plasma optical emission spectrometer (ICP-OES). By thermal gravimetric analysis (Fig. S6), the curves of three RZnHCF samples with different shapes were almost overlapped and the mass of water in RZnHCF was negligible. An intriguing effect of shape on the color of RZnHCF powders was found. Irregular RZnHCF is bright yellow, whereas shape-controlled RZnHCFs show green yellow color. Their UV-Vis diffuse reflectance spectra (Fig. S5b) reveals that the cuboctahedron RZnHCF (C-RZnHCF), truncated octahedron RZnHCF (T-RZnHCF) and octahedron RZnHCF (O-RZnHCF) can respectively absorb visible light with a wavelength at 561 nm, 559 nm and 550 nm. For the irregular RZnHCF, the absorption edge is drastically shifted to 588 nm, implying that regular shape may affect the frequency of the stretching mode of the cyano anion in RZnHCF.

**Figure 5:**
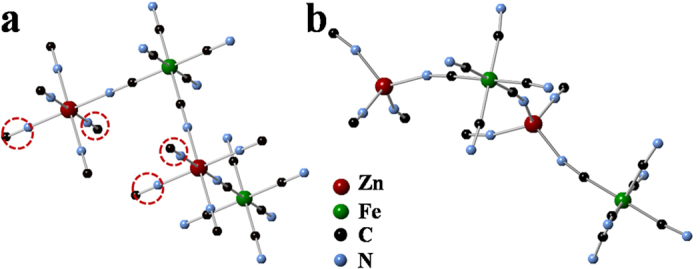
(**a**) Coordination environments for Zn and Fe atoms in cubic structure. The red dashed circles represent O atoms from water molecules, and the octahedral units, [Fe(C)_6_] & [ZnN_4_(O)_2_], behave as rigid blocks. (**b**) Coordination environments for Zn and Fe atoms in rhombohedral phase. The octahedral unit, [Fe(C)_6_], behaves as a practically rigid block. The tetrahedron ZnN_4_ is slightly distorted.

To clarify the process of structure transformation from cubic to rhombohedral, it is important to recognize the diversity of the cubic structure of PBAs. The general formula of PBAs is A_x_M[P(CN)_6_]_y_•nH_2_O, in which A is alkali metals and M & P are transition metals. When the ratio of M/P is 1, there are no vacancies in the cubic structure. If the ratio of M/P is not equal to 1, such as Fe_4_[Fe(CN)_6_]_3_^43^ or Cu_2_Fe(CN)_6_^44^, the [P(CN)_6_]^z−^ vacancies will appear in the cubic structure. In the cubic phase Zn_3_[Fe(CN)_6_]_2_ •xH_2_O we synthesized, the M/P ratio is 3/2, similar to that of Cu_3_[Co(CN)_6_]_2_^45^, so the [Fe(CN)_6_]^3−^ vacancies are presented as shown in Fig. S7a. According to previous study^46^, only 2/3 of the [Fe(CN)_6_]^3−^ atomic sites are occupied to achieve the electroneutrality with the face-centered cubic framework of Zn atoms. 1/3 of the [Fe(CN)_6_]^3−^ atomic sites are left as vacancies in the crystal structure, which are nominally occupied by water molecules to pair with the uncoordinated Zn atoms. The other uncoordinated water molecules are locted in the cages of cubic structure, as seen in Fig. S7b. However, removal of allthese water molecules from cubic phase ZnHCF will lead to the instability of the crystal structure, giving rise to a structural transformation as observed in our experiments. This structural transformation process is illustrated in Fig. 5a, b. Only part of the unit cell is presented in the figures for clarity. After drying of the Zn_3_[Fe(CN)_6_]_2_ •xH_2_O sample at 70 °C, coordinated water in the crystal is removed, resulting in the transformation of octahedrally coordinated ZnN_4_(O_2_) (Fig. 5a) to be tetrahedrally coordinated ZnN_4_ (Fig. 5b). Thus, the N-Zn-N bond in the dehydrated ZnHCF can no longer sustain its original linearity state in cubic phase ZnHCF, but forms a bond angle of ~108°, typical of tetrahedral coordination. The [Fe(CN)_6_] units, however, still approximately preserve their original geometry. Consequently, the change of the coordination condition of Zn leads to the transformation from the cubic phase to the rhombohedral phase as shown in Fig. S8. This coordination configuration in RZnHCF results in the formation of large ellipsoidal cavities (Fig. S9), in which the inserted zinc ions can occupy. This structure transformation can be considered as a distortion rather than reconstruction of the internal atoms, therefore the original morphologies of ZnHCF particles which are typical of cubic symmetry are still preserved in RZnHCF samples. Nevertheless, the structural distortion changes the atomic arrangements on the surfaces of the polyhedral particles. For convenience, the orientations of dehydrated RZnHCF crystals are named F111 and F100 to correlate with the {111} and {100} surfaces on fcc polyhedrons whose morphologies are preserved after structure transformation, while their structures demonstrated in Fig. S10, are totally different from {111} and {100} crystal planes in cubic ZnHCF.

### Morphology Effect on Electrochemical Performance

**Figure 6:**
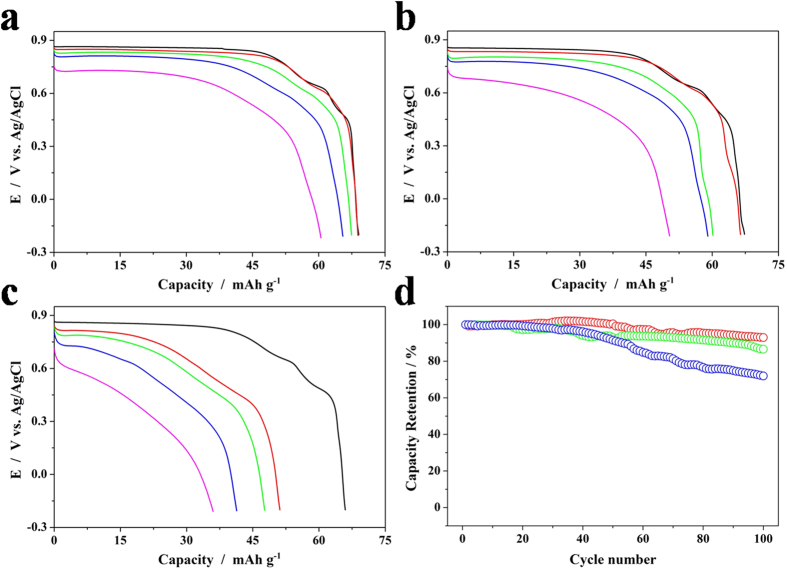
Electrochemical properties of shape-controlled RZnHCFs in 3M ZnSO_4_ electrolytes. The discharge curves at the rate of 60 mA g^−1^ (black), 300 mA g^−1^ (red), 600mA g^−1^ (green), 1200 mA g^−1^ (blue) and 3000 mA g^−1^ (magenta) of (**a**) C-RZnHCF, (**b**) T-RZnHCF and (**c**) O-RZnHCF based on the initial discharge capacity. (**d**) Cycle life test of shape-controlled RZnHCFs at 300 mA g^−1^. Red, green and blue circles represent C-RZnHCF, T-RZnHCF and O-RZnHCF, respectively.

The electrochemical performance of the dehydrated RZnHCF samples with different shapes was then studied by three-electrode flooded cells. Figure S11 displays the cyclic voltammograms (CVs) of shape-controlled RZnHCF at a scan rate of 2 mV s^−1^ in 3 M ZnSO_4_ electrolytes over wide potential ranges, which indicates that the electrochemical reactions are the same accounting for the Fe^2+^/Fe^3+^ redox couple. Figure 6a–c present the first discharge capacities of C-RZnHCF, T-RZnHCF and O-RZnHCF, respectively, at various discharge rates from 60 mA g^−1^ to 3000 mA g^−1^. At 60 mA g^−1^ rate C-RZnHCF, T-RZnHCF and O-RZnHCF deliver similar discharge capacities of 69.1, 67.3 and 66.0 mAh g^−1^, respectively. With the increase of the discharge rate, the difference of the discharge capacity between three samples becomes increasingly apparent. The C-RZnHCF exhibits discharge capacities of 68.9, 67.4, 65.5 and 60.5 mAh g^−1^ at 300, 600, 1200 and 3000 mA g^−1^ rate, respectively, while the corresponding capacities of T-RZnHCF are only 66.4, 60.2, 59.0 and 50.3 mAh g^−1^, respectively. The discharge values of O-RZnHCF at higher rates are even lower (51.1, 47.7, 41.3 and 36.0 mAh g^−1^, respectively). Therefore, C-RZnHCF performs the best rate capability among all three samples, and O-RZnHCFthe worst. Although the O-RZnHCF particles are smaller than T-RZnHCF particles, O-RZnHCF performs worse rate capability than T-RZnHCF, implying that the shape of RZnHCF compounds is more critical for the rate performance than their dimensions. Cycling lives of shape-controlled RZnHCF is also evaluated at 300mA g^−1^ rate, and the results are shown in Fig. 6d. After 100 cycles, the capacity retention for C-RZnHCF, T-RZnHCF and O-RZnHCF was 93.0%, 86.6% and 72.0%, respectively. Again, C-RZnHCF excels other two samples in the term of cyclic stability.

As all three RZnHCF samples have the same composition and structure, their difference on the electrochemical properties thus is ascribed to their different particle shape. As mentioned above, the particles of C-RZnHCF possess the largest areal proportion of surface F100, T-RZnHCF the second, and O-RZnHCF the smallest (capped all by F111). Comparing with the electrochemical data, it is reasonable to deduce that the charge/discharge performance of RZnHCF is positively correlated with the area of F100. Figure S12 shows the atomic arrangements along the surface oreintations of polyhedral RZnHCF samples. Thus, it is anticipated that F100 surfaces whose orientations are aligned to zinc ions diffusion channels facilitate high discharge rate capabilities, which is confirmed by impedence measurements (Fig. S13). The low frequency region in the electrochemical impedance spectra (EIS, Fig. S13a) corresponds to the zinc-ion diffusion process within the electrodes. Figure S13b shows the relationship between Z_re_and ω^−1/2^ in the low frequency region, where ω is the angular frequecy (ω = 2πf). The low slope indicates good zinc-ion kinetics in the electrode materials. The C-RZnHCF shows the lowest slope among all three samples, suggesting best zinc-ion kinetics. It is anticipated that the less dense atomic arrangements along the F111 directions are more liable to interact with the electrolyte and thus accelerate the sample dissolution. So the C-RZnHCF sample with less F111 orientations in the surface areas will present a better cycling performance. In conclusion, the shape of RZnHCF particles with more surface areas with F100 orientations will be benficial to enhance the electrochemical performance. The similar influence of surface orientations on the ions diffusion and sample dissolution has been observed in other cathode materials^33^.

### Zinc-ion Battery Properties

**Figure 7:**
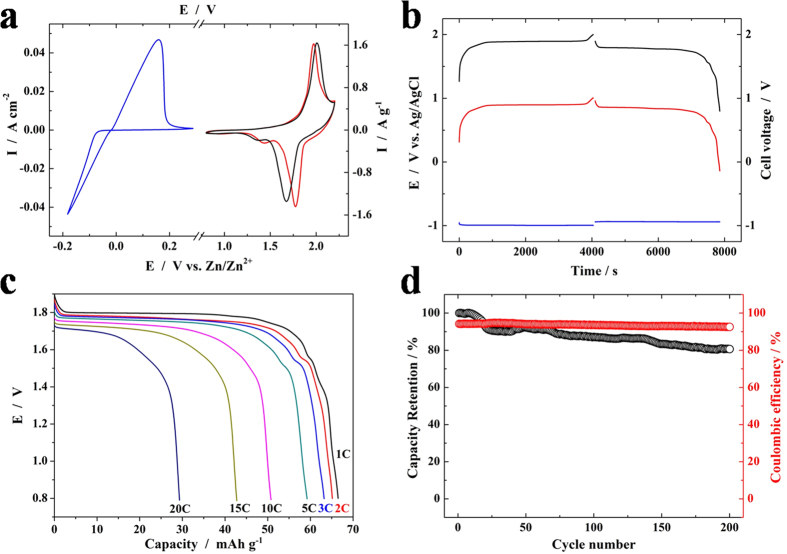
(**a**) CVs at a scan rate of 2 mV s^−1^ and (**b**) GCD profiles at 60 mA g^−1^ for the anode (blue), the cathode (red) and the full cell (black) in 3 M ZnSO_4_ aqueous electrolytes. (**c**) Discharge curves of the cell at different rates. (**d**) Cycle life tests at the current density of 300 mA g^−1^.

Because of its better electrochemical performance than other two RZnHCF samples, we chose C-RZnHCF as the cathode and zinc as the anode to manufacture the aqueous zinc-ion battery. The typical CV curves of two individual electrodes along with the full cell at a scan rate of 2 mV s^−1^ are displayed in Fig. 7a. The deposition and dissolution of zinc at anode occurs at around 0V vs. Zn/Zn^2+^. More details about the structure and morphology evolution during charge/discharge processes and electrochemical properties of zinc anode are exhibited in the supporting information (Fig. S14, S15, S16 and S17). All the evidence confirms that zinc anode is stable during cycling in the zinc-ion battery.Meanwhile, the distinctive reversible redox peaks of cathode appears at ca. 1.77 V and 1.97 V vs. Zn/Zn^2+^. The full cell exhibits a similar pair of redox peaks as the cathode, appearing at ca. 1.68 V and 2.00 V vs. Zn/Zn^2+^. The galvanostatic charge and discharge curves of the full cell under a current density of 60 mA g^−1^ between 0.8 V and 2.0 V are shown in Fig. 7b. It delivers a discharge capacity of 66.5 mAh g^−1^ based on the mass of active cathode materials (60.7 mAh g^−1^ based on the total mass of active electrode materials). And it has a flat discharge voltage plateau at ca. 1.8 V with an average operating voltage of ca. 1.73 V, delivering a specific energy density of 105 Wh kg^−1^ based on the total mass of active electrode materials(see Fig. S18 for details). The discharging voltage profiles at various current densities are displayed in Fig. 7c. Based on the mass of active cathode materials, it delivers the discharge capacities of 66.5, 65.1, 63.2, 59.2, 50.8, 42.7 and 29.3 mAh g^−1^ at 60, 120, 180, 300, 600, 900 and 1200 mA g^−1^ rate, respectively, showing good rate capability. The discharge capacity retains 80% of the maximum value after charged and discharged at 300 mA g^−1^ in the 3M ZnSO_4_ aqueous electrolyte for 200 cycles (Fig. 7d), indicating that the cell also possesses excellent cyclic stability. After 50 charge-discharge cycles in the 3M ZnSO_4_ electrolyte, the XRD pattern of C-RZnHCF electrode keeps the same with the original one (Fig. S19), further suggesting the electrochemical stability of C-RZnHCF.

### Reversible Zinc-ion Intercalation

**Figure 8:**
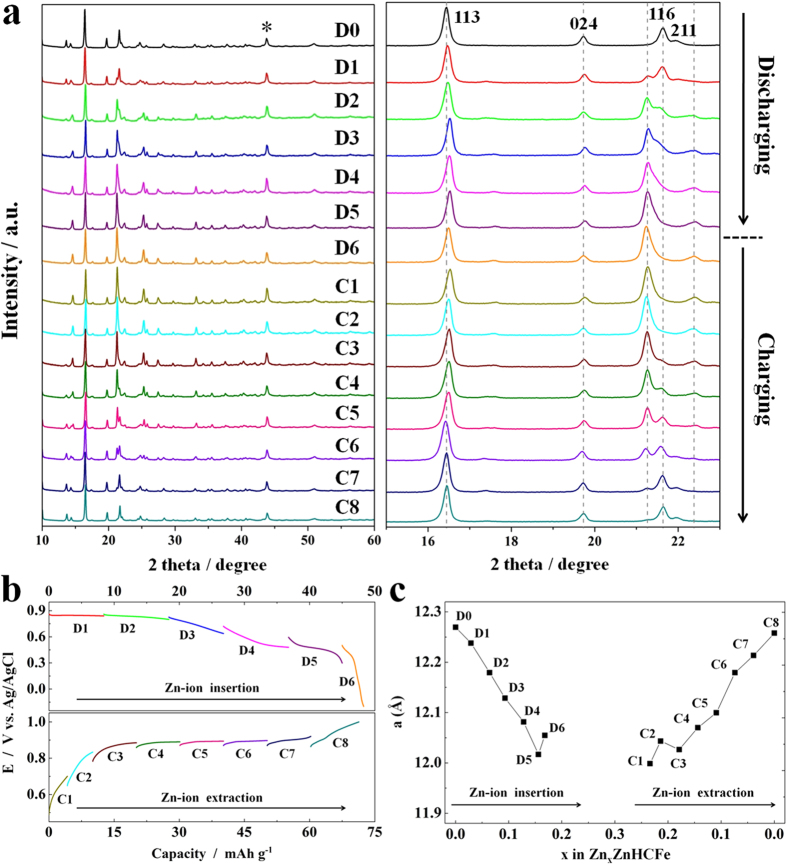
(**a**) *Ex situ* XRD patterns of the C-RZnHCF electrode at various discharge and charge states in the first cycle. D0 is the original one before electrochemical measurement. (**b**) Typical discharge/charge profiles of C-RZnHCF electrodes obtained at corresponding discharge/charge stages captured for *ex situ* XRD measurement. For example, after the discharge stage of D1, the electrode was characterized by XRD to get the D1 XRD pattern. (**c**) Changes of the value of lattice parameter (**a**) during Zn-ion insertion and extraction.

The investigation of the intercalation mechanism for divalent Zn^2+^ in RZnHCF was conducted in a three-electrode flooded cell with an C-RZnHCF working electrode, a Pt counter electrode and an Ag/AgCl reference electrode, and *ex situ* XRD patterns (Fig. 8a) of the C-RZnHCF electrode at different discharge/charge states of the first cycle in the aqueous 3 M ZnSO_4_ electrolyte along with corresponding discharge/charge curves (Fig. 8b) were measured. Asterisks (*) in Fig. 8a indicates the XRD peaks of the iron grid reference, which is used to calibrate lattice parameters of the same C-RZnHCF sample at different discharge/charge states. During the Zn^2+^ insertion process, the position of the strongest XRD peak (113) shifts slightly toward higher angles. The (104) and (110) peaks disappear and one peak near (110) peak appears with discharging. The distinct variation of XRD patterns occurs at the (116) and (211) peaks around 2theta of 22°. During discharging these two peaks split. (116) peak moves to lower angles and (211) peak moves to higher angles. In addition, the intensity of moved (116) peak increases with the insertion of zinc ions. At the end of discharging, its intensity is even higher than the strongest peak (113) of the pristine XRD pattern. During the charging process, the XRD pattern evolves in a reverse way comparing with that in the discharging process, and finally recovers to the original state before discharging, indicating that the structural change along with the insertion/extraction of Zn^2+^ is highly reversible. Figure S20 displays the *ex situ* XRD patterns measured in the second cycle, which is the same as those in the first one and further confirms reversible zinc-ion intercalation mechanism.

In Fig. 8c, the value of the unit cell parameter, a, for the C-RZnHCF, determined from the XRD patterns in Fig. 8a, is plotted vs. zinc-ion incorporation, x, in Zn_x_ZnHCFe. ZnHCFe represents the formula of C-RZnHCF, K_0.07_Zn[Fe(CN)_6_]_0.69_, determined by ICP and thermogravimetric analysis. Limited by the number of electrons available from Fe^2+^/Fe^3+^ redox couple, the same absolute charge, i.e., 0.6 Na^+^ or K^+^, or 0.3 Zn^2+^ is stored per formula unit of ZnHCFe. Thus the value of x can change from 0 to 0.3. The C-RZnHCF possesses a rhombohedral phase with space group of *R-3ch*, so the lattice parameter was calculated by Bragg equation and lattice parameter caculation formula of hexagonal system based on (113) and (024) peaks, which always exist during Zn^2+^ insertion/extraction. The original XRD pattern was refined by the Rietveld method in Maud software based on two peaks (113) and (024) (Fig. S21) to indentify the lattice parameter, a, which was close to our calculation of the original one, 12.270 Å. The lattice parameter monotonically decreases from 12.270 Å (x = 0) to 12.017 Å (x = 0.16) upon insertion of zinc ions and slightly increases to 12.055 Å at x = 0.17. In the successive discharge process (extraction of zinc ions), the a value then monotonically increases back to 12.261 Å when fully charged, almost the same as that in the original sample, which indicates high reversibility of Zn^2+^ intercalation in the RZnHCF framework. However, the detailed investigation of structural transformation of RZnHCF during zinc-ion insertion/extraction needs more experimental evidence, which is currently underway in our lab.

## Discussion

To investigate the influence of morphology on the electrochemical performance, we demonstrate a facile and general procedure for preparation of single-crystalline ZnHCF polyhedrons by a simple co-precipitation method without capping agent. However, Three cubic phase ZnHCF samples with cubooctahedral, truncated octahedral or octahedral shapes are obtained at room temperature. Moreover, due to the lost of coordinated water, the structural transformation from cubic phase to rhombohedral phase occurs when the ZnHCF samples are dried at elevated temperature, whereas the apparent polyhedral shapes are still well maintained.

Meanwhile, we found that the electrochemical performance of RZnHCF is strongly dependent on their shapes. As a result, RZnHCF with cuboctahedron morphology exhibits better performance in the terms of rate capability and cyclic stability comparing with other two samples, which implies that the diffusion of zinc ions and dissolution of active materials is closely correlative with the structure of surface orientations. While this study is devoted to cathodes based on ZnHCF, a kind of PBAs, with controlled morphology, it seems that the present findings can be further generalized. Therefore, controlling the shape of PBAs with special facets exposed is believed to be an important means to improve their electrochemical performance.

Becuase of the significantly improved performance derived from the fast zinc-ion diffusion and limited dissolution of sample, the C-RZnHCF was chosen as the cathode combined with zinc as the anode to manufacture the aqueous zinc-ion battery in 3M ZnSO_4_ electrolytes. Consequently, the battery delivers a high energy density of 104 Wh kg^−1^ with an average operating voltage of ca. 1.73 V. Finally, *ex situ* X-ray diffraction techniqueprovides conclusive evidence of the highly reversible zinc-ion intercalation in the rhombohedral framework, which is helpful to understand the intercalation chemistry based on ZnHCFs.

## Methods

### Synthesis of Polyhedral ZnHCF

Polyhedral ZnHCF particles were obtained by a simple co-precipitation method at room temperature in the absence of surfactants. Typically, 2 mmol zinc sulfate (ZnSO_4_ •7H_2_O, Sinopharm Chemical Reagent Co., Ltd, Shanghai, China) was dissolved in 200 ml deionized water to obtain solution A, and 2 mmol potassium ferricyanide (K_3_Fe(CN)_6_, Aladdin Industrial Inc, Shanghai, China) was also dissolved in 200 ml deionized water to obtain solution B. Then, solution A and solution B were simultaneously added into an empty beaker under vigorous stirring at room temperature, and the addition time of solution A and B was controlled. After stirring for 24 hours at room temperature, the suspensions were left to stand for 12hours. Then, the obtained solids were filtered, washed with deionized water, and dried at room temperature to obtain cubic ZnHCF. For preparing the cathode electrode, the as-synthesized ZnHCF solids were further dried at 70 °C for 12 hours. The shapes of the polyhedral ZnHCF could be tuned by adjusting the addition time of solution A and B. When solution A and B were poured into the beaker within a total addition time of several seconds, ZnHCF with cuboctahedron shape was obtained. If the reaction solutions were dropped by plastic pipettes within a time span of ~20 minutes, truncated octahedral ZnHCF particles were prepared. And the octahedron shape particles could be formed by further elongating the addition time to ~2 h, using a peristaltic pump. The synthesis method of irregular ZnHCF has been introduced in our previous work^30^.

### Synthesis of MnHCF, CoHCF and AgHCF with Regular Shapes

2 mmol manganese sulfate, cobalt sulfate or silver nitrate (MnSO_4_ •H_2_O, CoSO_4_ •7H_2_O or AgNO_3_, purchased from Sinopharm Chemical Reagent Co. or Aladdin Industrial Inc, Ltd, Shanghai, China) was dissolved in 200 ml deionized water to obtain solution A, and 2 mmol potassium ferricyanide (K_3_Fe(CN)_6_, Aladdin Industrial Inc, Shanghai, China) was also dissolved in 200 ml deionized water to obtain solution B. Then, solution A and B were poured into the beaker under vigorous stirring in several seconds. After agitation and aging for 12 h, the products were filtered and washed by deionized water. Finally, it was dried at 70 °C.

### Materials Characterization

Powder X-ray diffraction patterns were performed on an AXS D8 Advance diffractometer (Cu Kα radiation, λ = 1.5406 Å; receiving slit, 0.2 mm; scintillation counter, 40 mA; 40 kV) from Bruker Inc. *Ex-situ* XRD diffraction patterns of cathode electrodes at various charge/discharge states were collected on a Bruker D8 diffractometer operating in Bragg-Brentano geometry with Cu Kα radiation. The morphology of particles was observed by a Hitachi S-4800 field emission scanning-electron microscope at an accelerating voltage of 8 kV. The UV-Vis diffusivereflectancespectra were obtained by Perkin Elmer Lambda 950 spectrometer. Thermal gravimetric analysis was performed on a Pyris Diamond thermogravimetric/differential thermal analyzer (Perkin-Elmer) to analyze the water content in three polyhedral ZnHCFs powders. The K: Zn: Fe ratios of shape-controlled RZnHCFs were determined by inductively coupled plasma optical emission spectrometer (Perkin Elmer Optima 2100 DV). The chemical formulas of C-RZnHCF, T-RZnHCF and O-RZnHCF was found to be K_0.07_Zn[Fe(CN)_6_]_0.69_, K_0.08_Zn[Fe(CN)_6_]_0.67_ and K_0.07_Zn[Fe(CN)_6_]_0.68_, respectively.

### Electrochemical Measurement

Electrochemical measurements were carried on Solartron 1470E multi-channel potentiostats using either a two-electrode or a three-electrode cell setup. Composite electrodes were cast on steel iron grid. The slurries were prepared by mixing active materials (75 wt%), Super P (15 wt%) and polyvinylidene fluoride (10 wt%) in N-methyl-2-pyrrolidinone. Finally, electrodes were dried at 80 °C for 12 h in air. Discs with diameter of 1.3 cm were cut for electrochemical tests. Mass loadings for the electrodes were determined by comparing the mass of the electrode with that of the original blank one. Typical electrode active material loading was ca. 8 mg/cm^2^. The electrode for *ex situ* X-ray measurement was prepared by extracting the cathode from the three-electrode cell and washing it with de-ionized water and drying at room temperature. For three-electrode setup, an Ag/AgCl electrode and Pt gauze were employed as reference and counter electrodes, respectively. For the two-electrode cell, the zinc anode was prepared by mixing Zn power, activated carbon and polyvinylidene fluoride to obtain the slurry at an appropriate viscosity, which was then coated on current collector, dried at 80 °C, and punched into discs of 1.3 cm in diameter. 3M aqueous ZnSO_4_ solution was used as the electrolytes.The mass ratio of active anode materials to active cathode materials (9 : 100) can be referred to our previous work^30^.

## Additional Information

**How to cite this article**: Zhang, L. *et al.* Morphology-Dependent Electrochemical Performance of Zinc Hexacyanoferrate Cathode for Zinc-Ion Battery. *Sci. Rep.* **5**, 18263; doi: 10.1038/srep18263 (2015).

## Supplementary Material

Supplementary Information
