# Supplementary material for: Morphology-Dependent Electrochemical Performance of Zinc Hexacyanoferrate Cathode for Zinc-Ion Battery
Source: Sci Rep. 2015 Dec 16;5:18263. doi: 10.1038/srep18263 (PMC4680913; doi:10.1038/srep18263)
Supplement: Supplementary Information [file srep18263-s1.doc]

Supplementary Information for “Morphology-Dependent Electrochemical Performance of Zinc Hexacyanoferrate Cathode for Zinc-Ion Batteries”

Leyuan Zhang, Liang Chen, Xufeng Zhou* and Zhaoping Liu*

Ningbo Institute of Materials Technology and Engineering, Chinese Academy of Sciences,Zhejiang 315201, P.R. China

*email: liuzp@nimte.ac.cn (Z.P.Liu), zhouxf@nimte.ac.cn (X.F.Zhou)


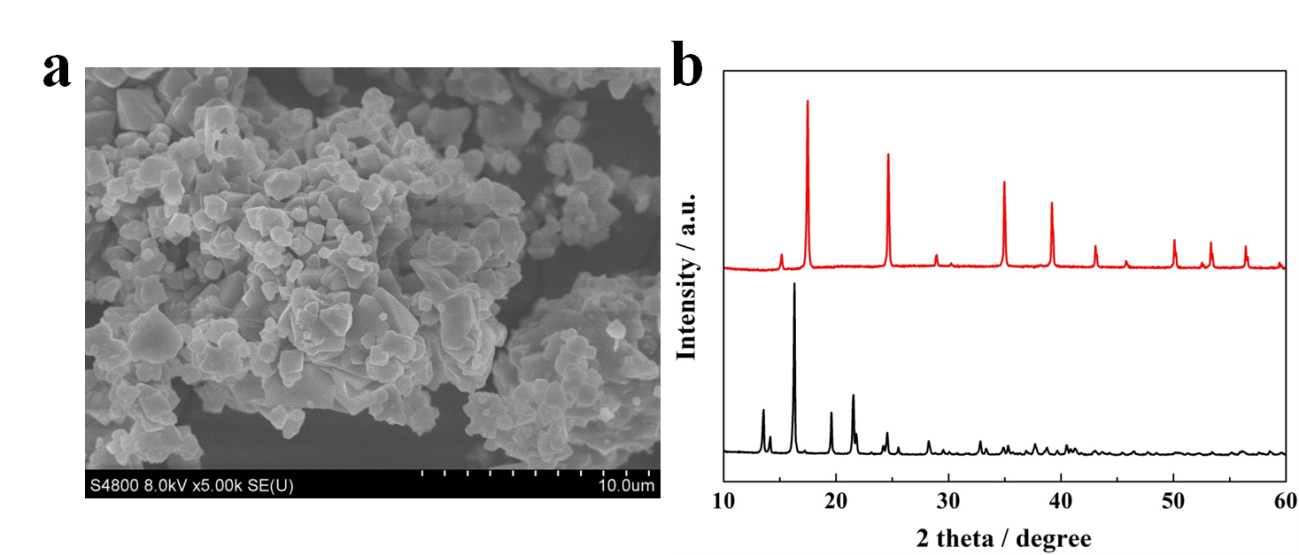


**Fig. S1**. (a) SEM image of irregular ZnHCF and (b) XRD patterns of irregular ZnHCF with cubic (red) and rhombohedral (black) phase.


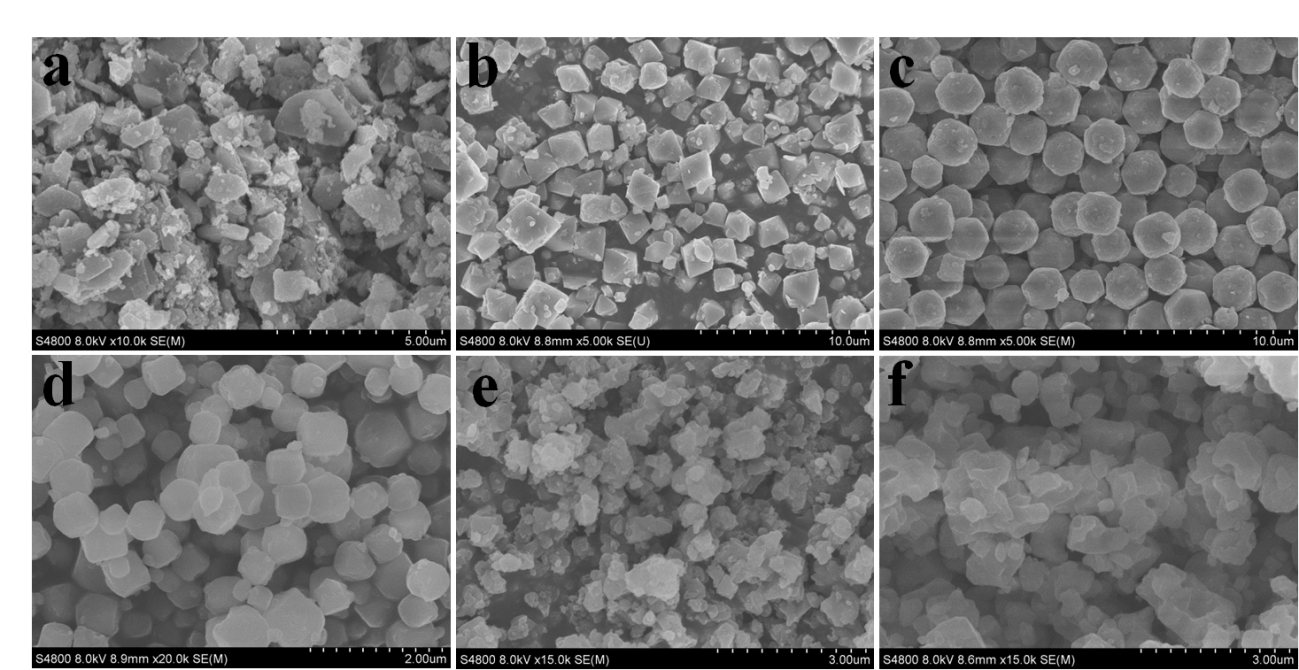


**Fig. S2**. SEM images of the ZnHCF solids synthesized with different reactant concentrations of (a) 0.001M, (b) 0.002M, (c) 0.005M, (d) 0.02M, (e) 0.05M and (f) 0.1M.


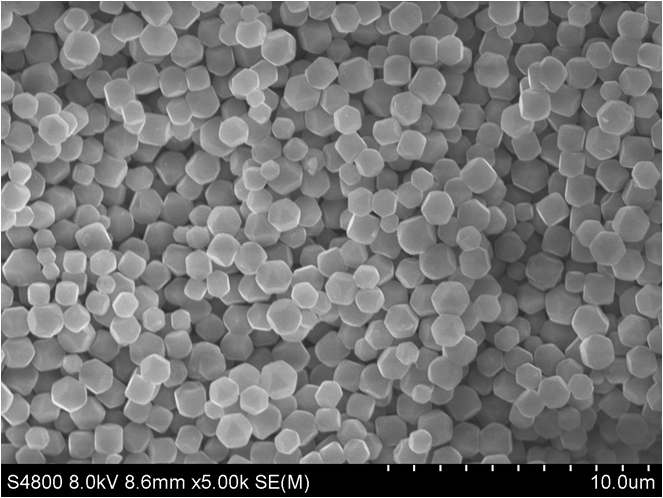


**Fig. S3**. SEM image of uniform ZnHCF crystals synthesized by controlling the reactant concentration to 0.01M without the process of agitation and age.


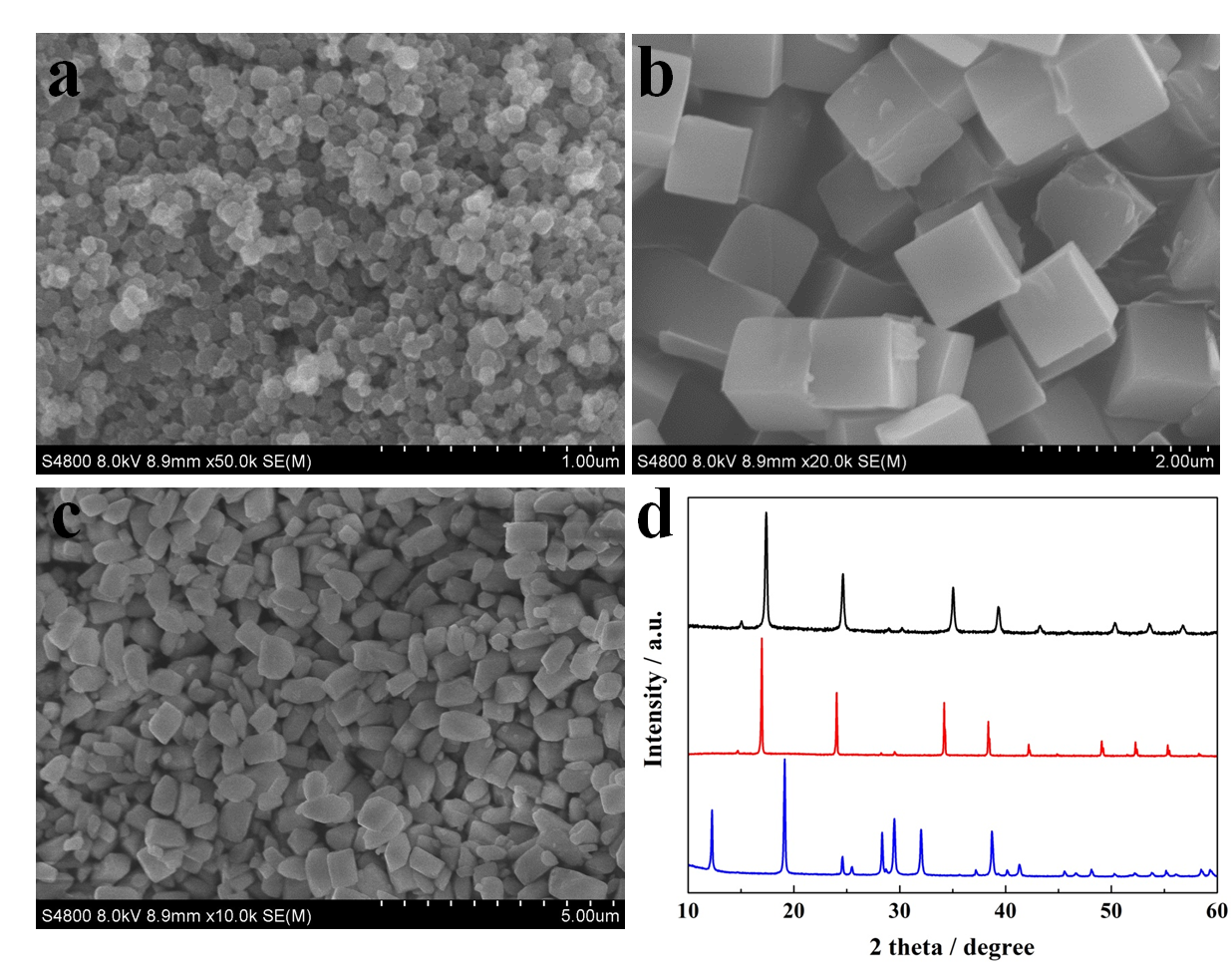


**Fig. S4**. SEM images of (a) CoHCF, (b) MnHCF and (c) AgHCF with regular shapes. (d) XRD patterns of CoHCF (black), MnHCF (red) and AgHCF (blue).


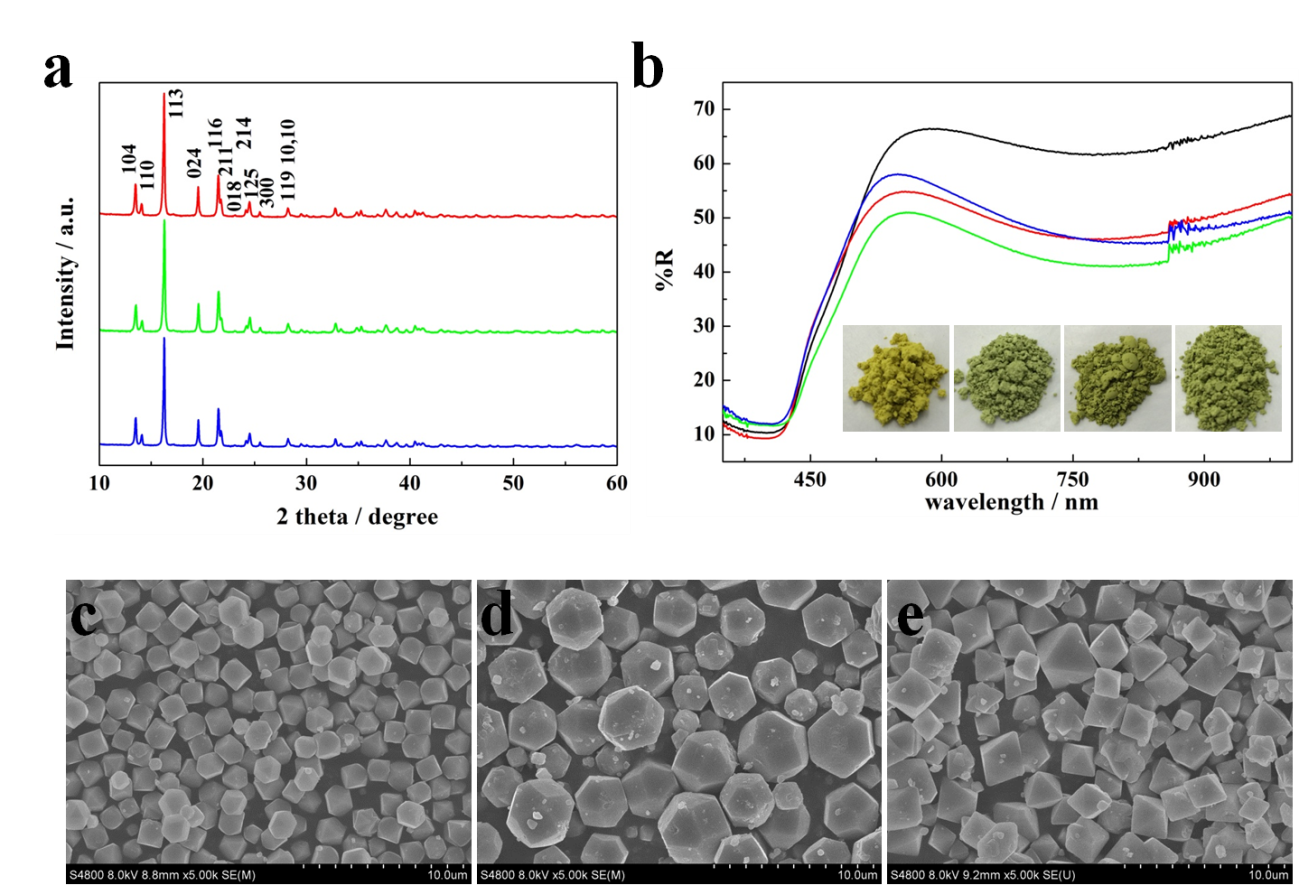


**Fig. S5**. (a) XRD patterns of C-RZnHCF (red), T-RZnHCF (green) and O-RZnHCF (blue); (b) UV-Vis diffuse reflectance spectra and photos (insets from left to right) of irregular RZnHCF (black), C-RZnHCF (red), T-RZnHCF (green) and O-RZnHCF (blue); SEM images of (c) C-RZnHCF, (d) T-RZnHCF and (e) O-RZnHCF.


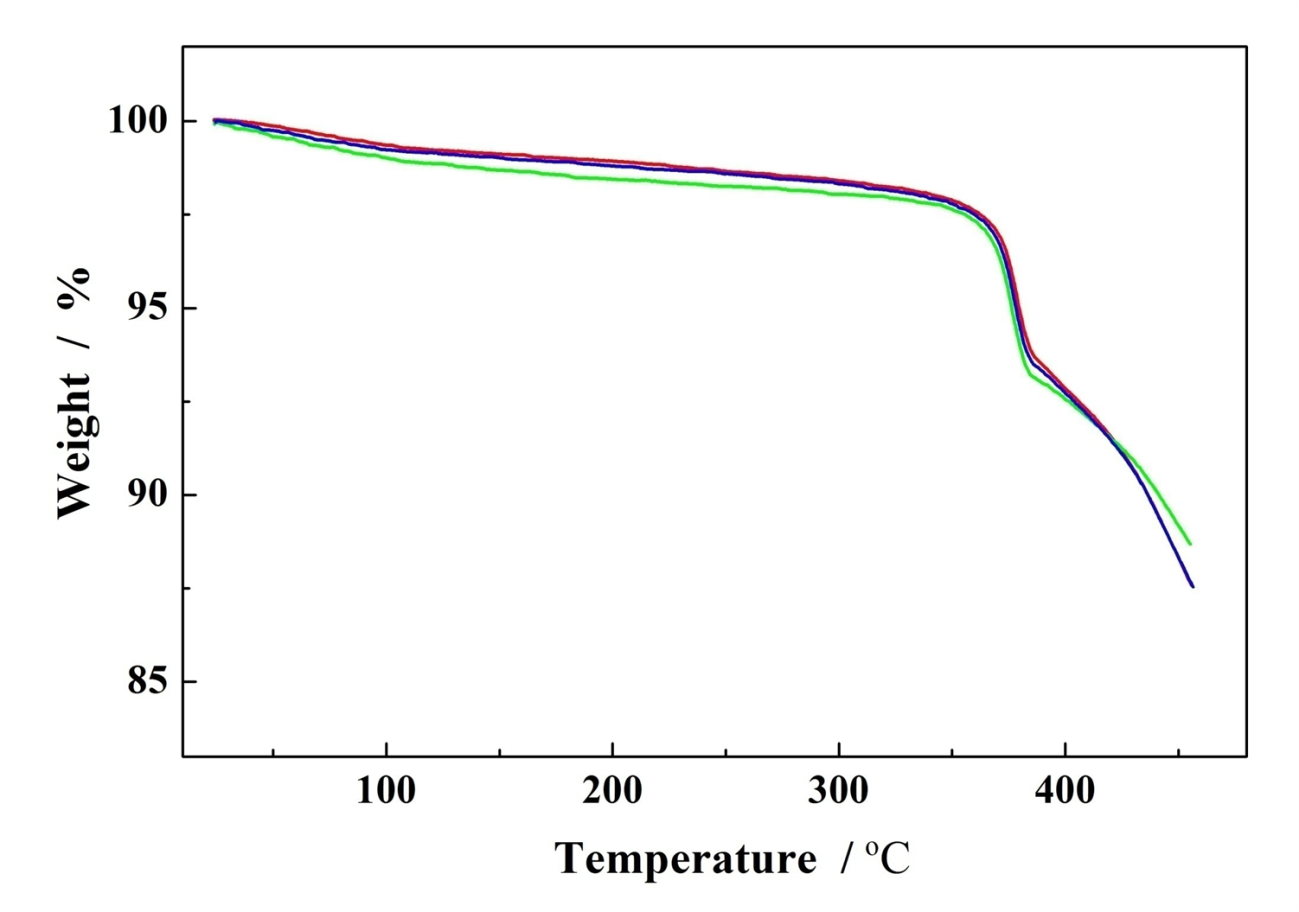


**Fig. S6**. TGA curves of C-RZnHCF (red), T-RZnHCF (green) and O-RZnHCF (blue).

**
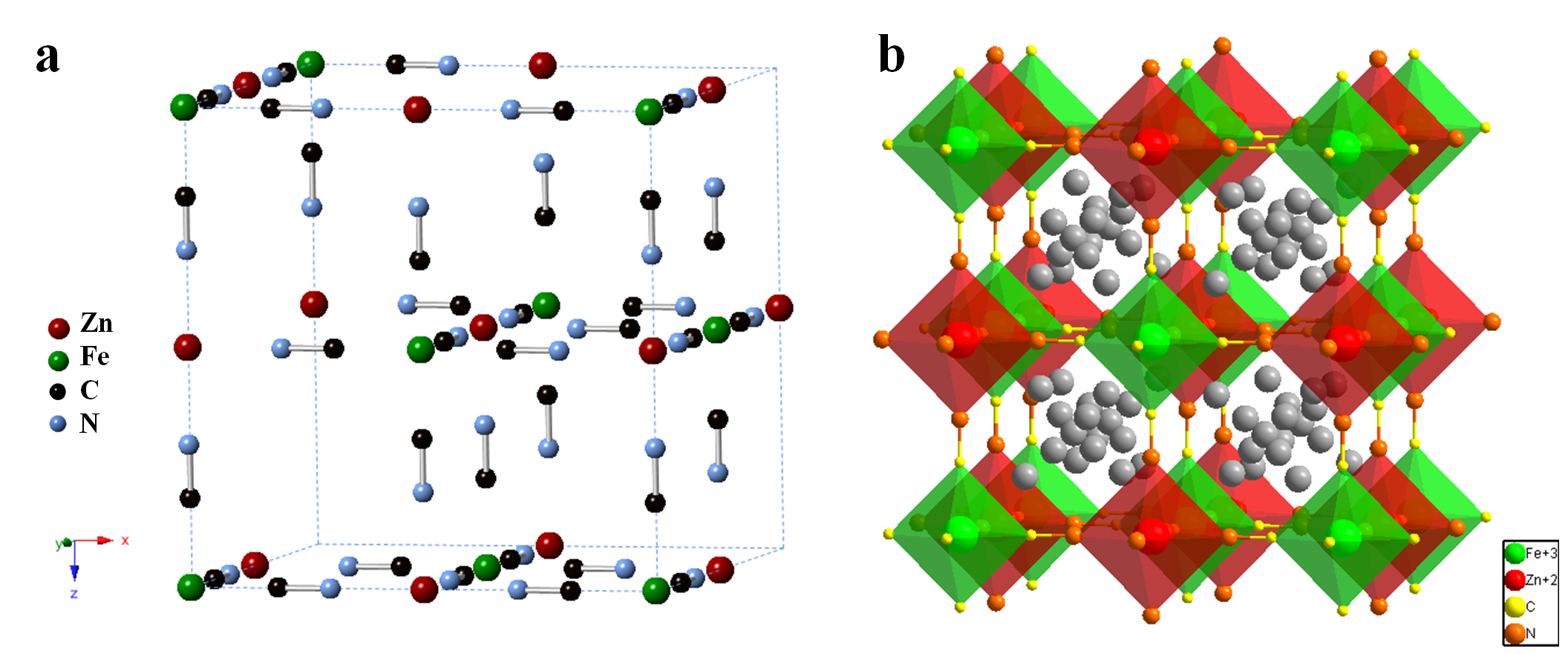
**

**Figure S7.** (a) The crystal structure of cubic Zn_3_[Fe(CN)_6_]_2_•xH_2_O, in which 1/3 of the [Fe(CN)_6_]^3-^ sites are occupied by water molecules. The water molecules are omitted for clarity.(b) The position of uncoordinated water molecules in cubic structure. The grey balls represent O atoms of H_2_O molecules.


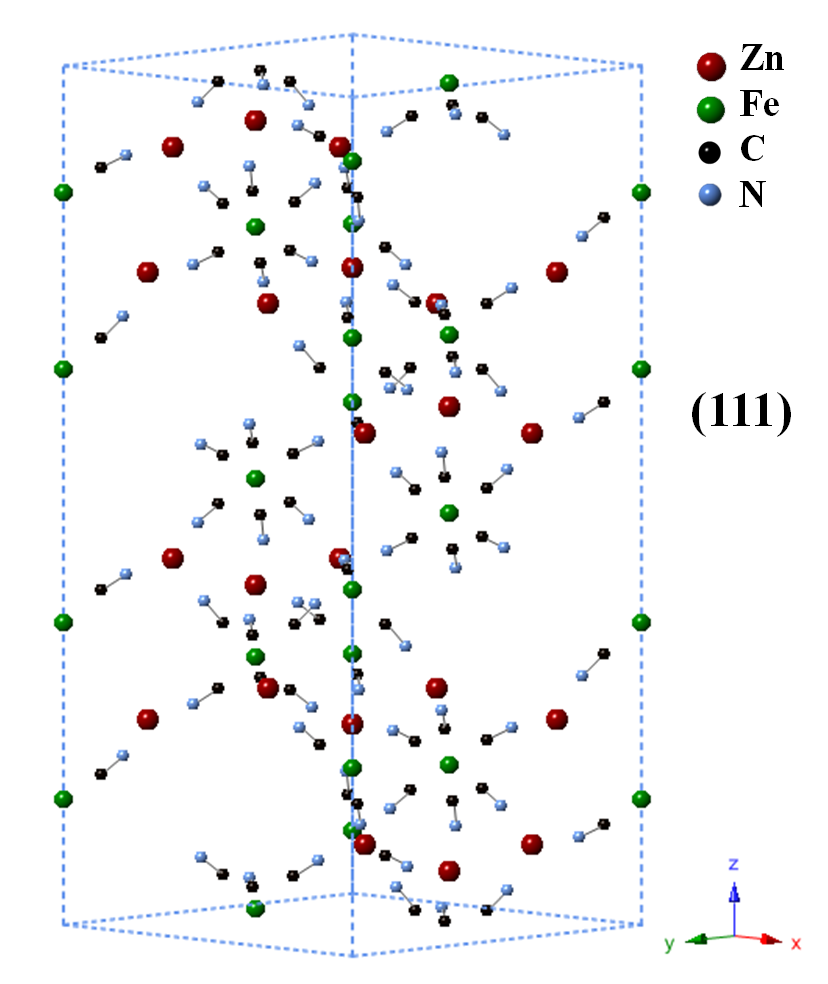


**Fig. S8.** Crystal structure of rhombohedral Zn_3_[Fe(CN)_6_]_2_ along crystal plane (111).


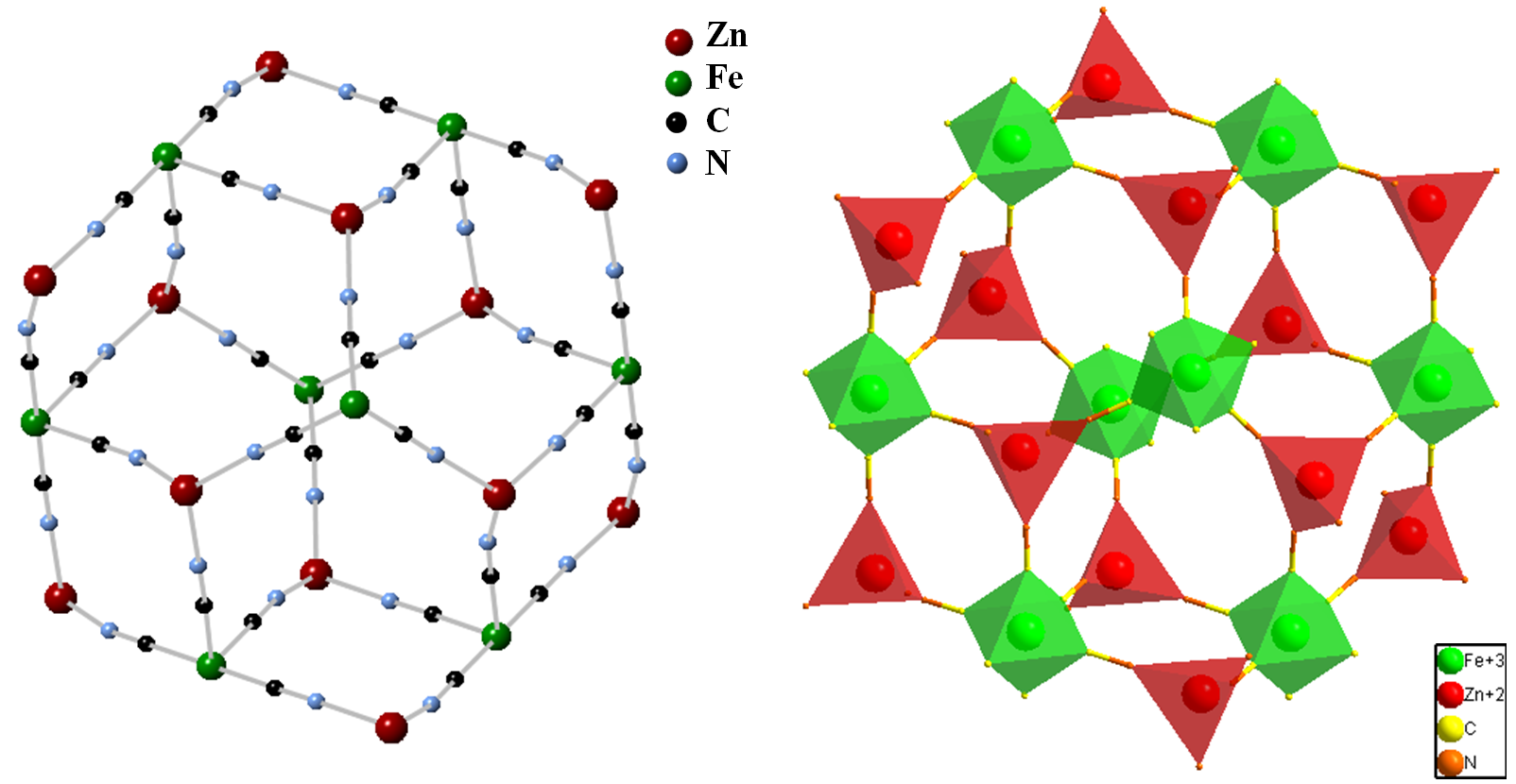


**Fig. S9.** An isolated cavity within the framework for the rhombohedral phase ZnHCF where 8 FeC_6_ octahedra and 12 ZnN_4_ tetrahedra are observed at the surface (view along the [001] direction).


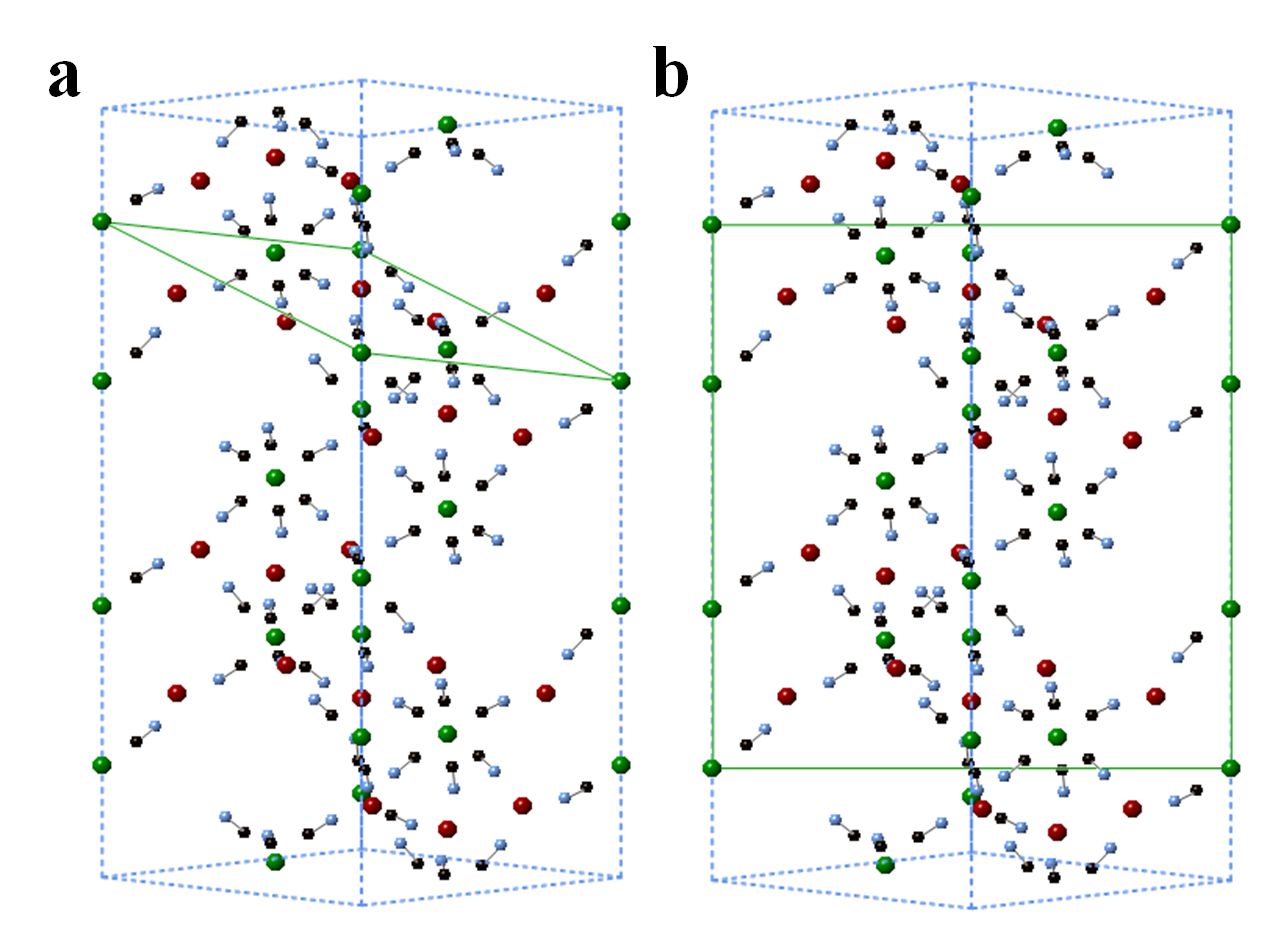


**Fig. S10**. A view of (a) F111 and (b) F100 (marked by solid lines) in the rhombohedral structure.


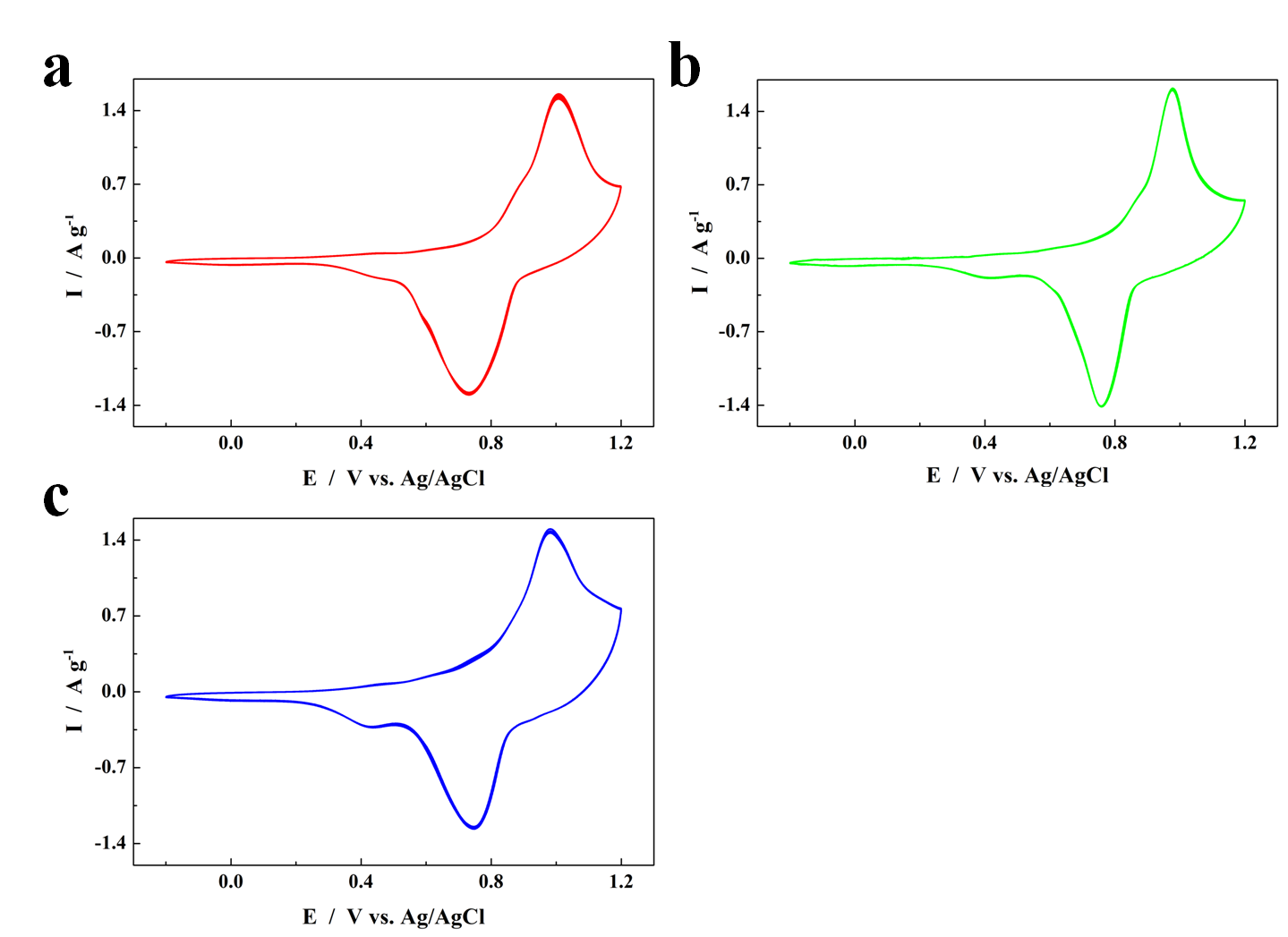


**Fig. S11.** CV curves of (a) C-RZnHCF, (b) T-RZnHCF and (c) O-RZnHCF in 3M ZnSO_4_ electrolytes during the latter 5 cycles of initial 10 cycles at a scan rate of 2 mV s^-1^.


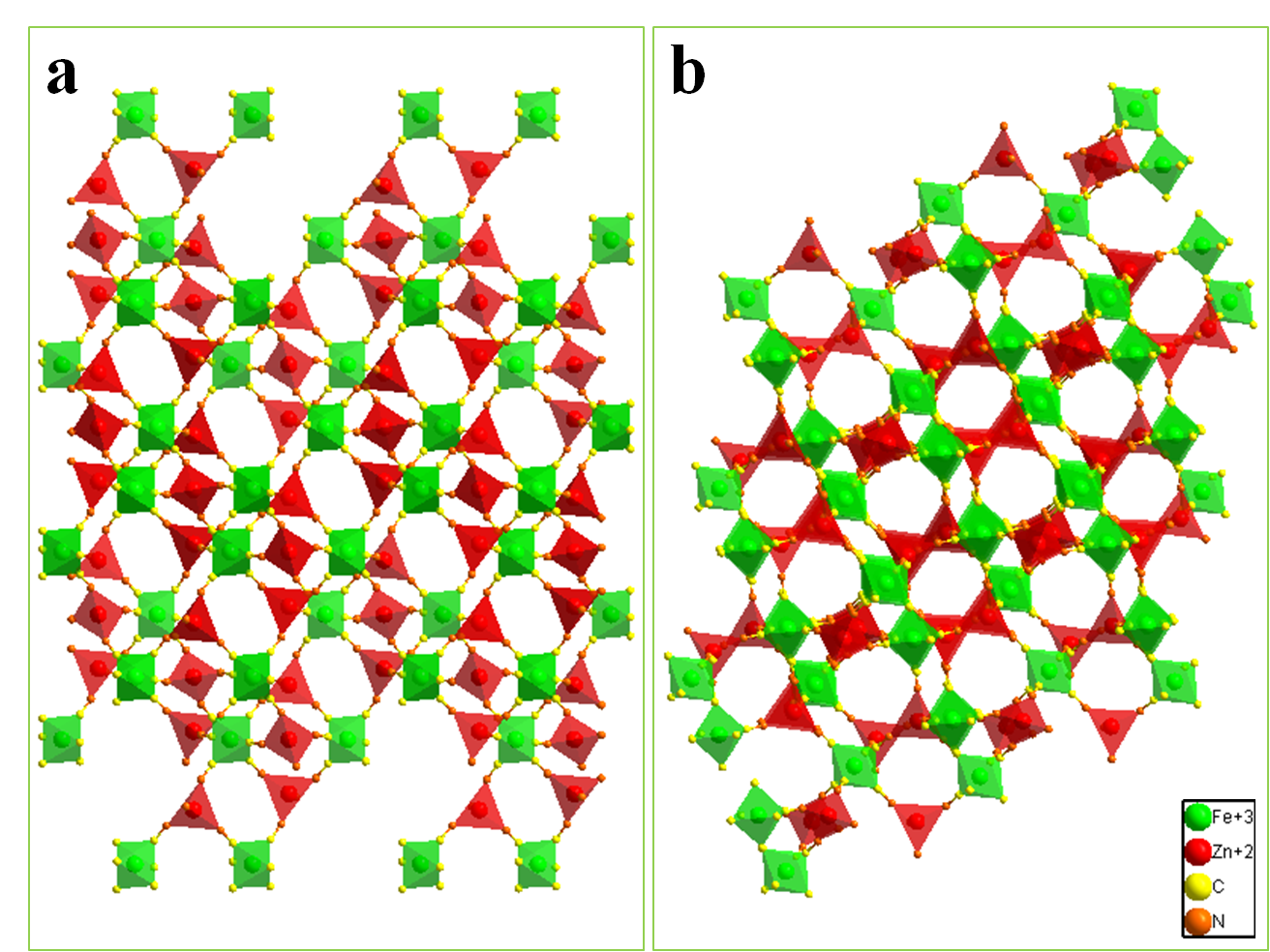


**Fig. S12**. An illustration of the atomic arrangements along different surface orientations of (a) F100 and (b) F111 in the rhombohedral structure. The view direction is perpendicular to the F100 and F111orientations.


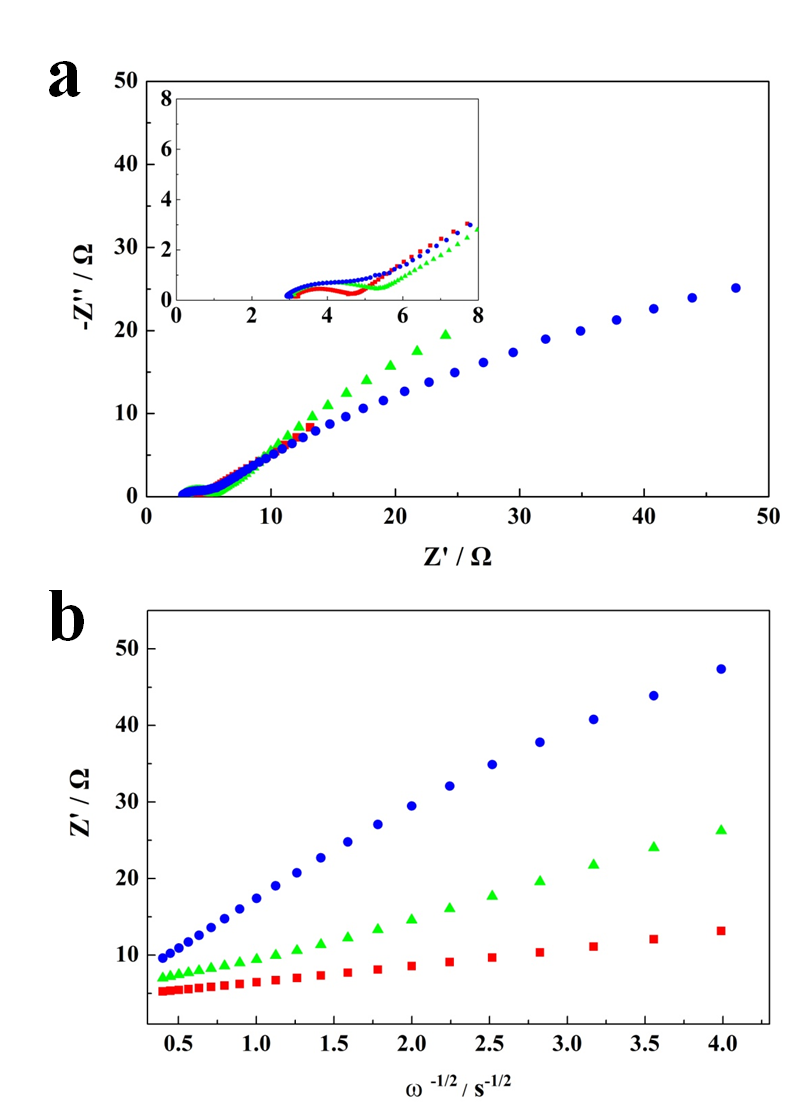


**Fig. S13.** (a) Electrochemical impedance spectra (EIS) and (b) relationship between Z＇and ω^-1/2^ in the low-frequency region of the C-RZnHCF (red), T-RZnHCF (green) and O-RZnHCF (blue).


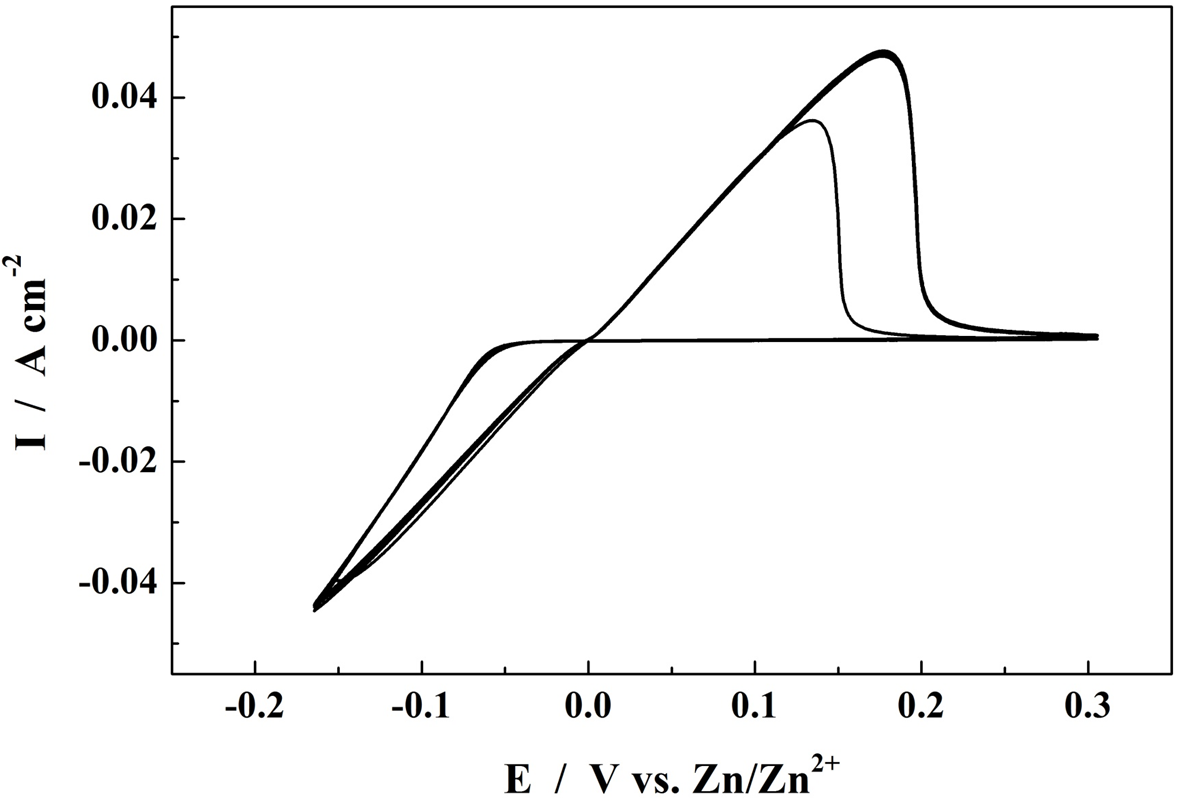


**Fig. S14.** CV curves of zinc deposition and stripping in 3M ZnSO_4_ electrolytes during the initial 10 cycles at a scan rate of 2 mV s^-1^. The CV curves are almost overlapped after the first cyle, suggesting that the deposition and stripping of zinc is quite stable during cycling.


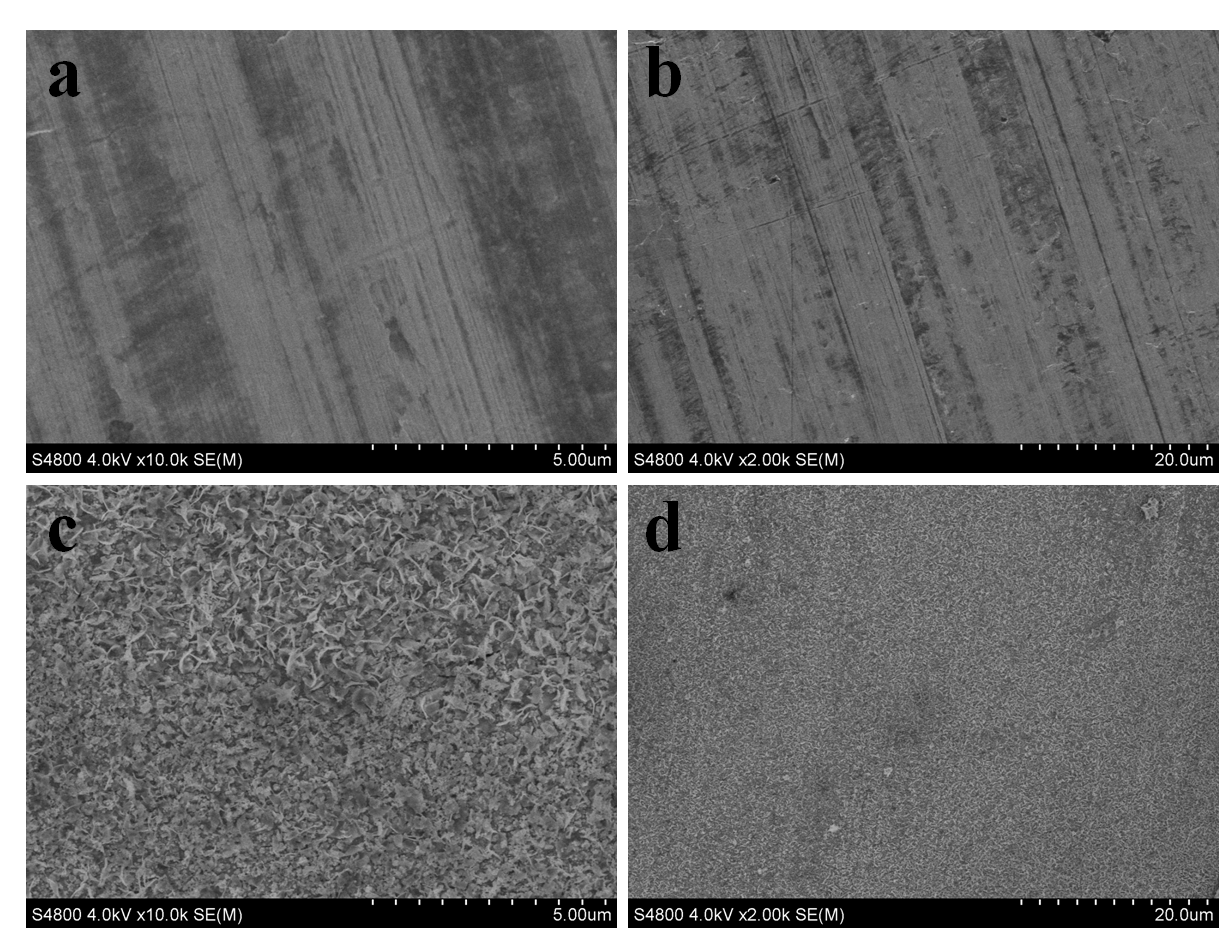


**Fig. S15.** SEM images of zinc foil anode before (a, b) and after 120 cycles (c, d) in the zinc-ion battery. Before charging and discharging, the zinc anode has a smooth surface. After cycling in the zinc-ion battery, a rougher layer was deposited on the surface but sharp zinc dendrite was not observed. Similar morphology change has also been observed in a previous report (Kang et al., *Angew. Chem. Int. Ed.* 2012*, 51*, 933).


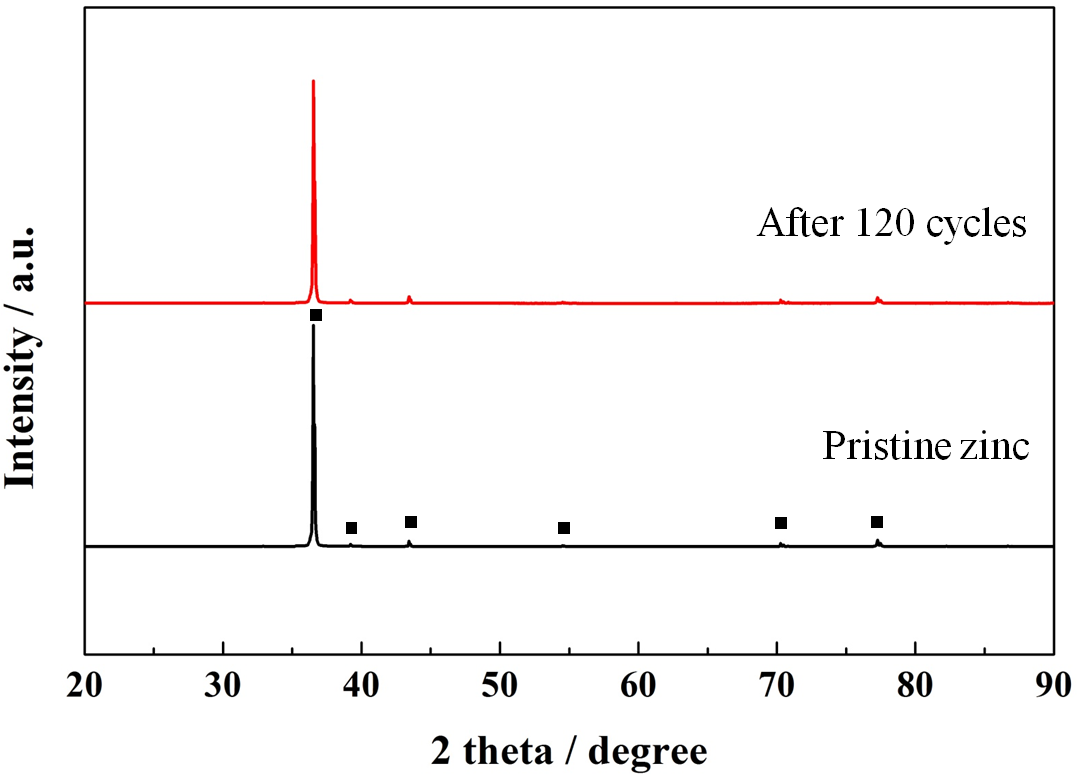


**Fig. S16.** XRD patterns of zinc film anode before and after 120 cycles. The idential patterns before and after cycling confirm that the structure of zinc anode after cycling is accordance to pure zinc. ZnO or Zn(OH)_2_ which always induce irreversible capacity did not form in the cycling process.


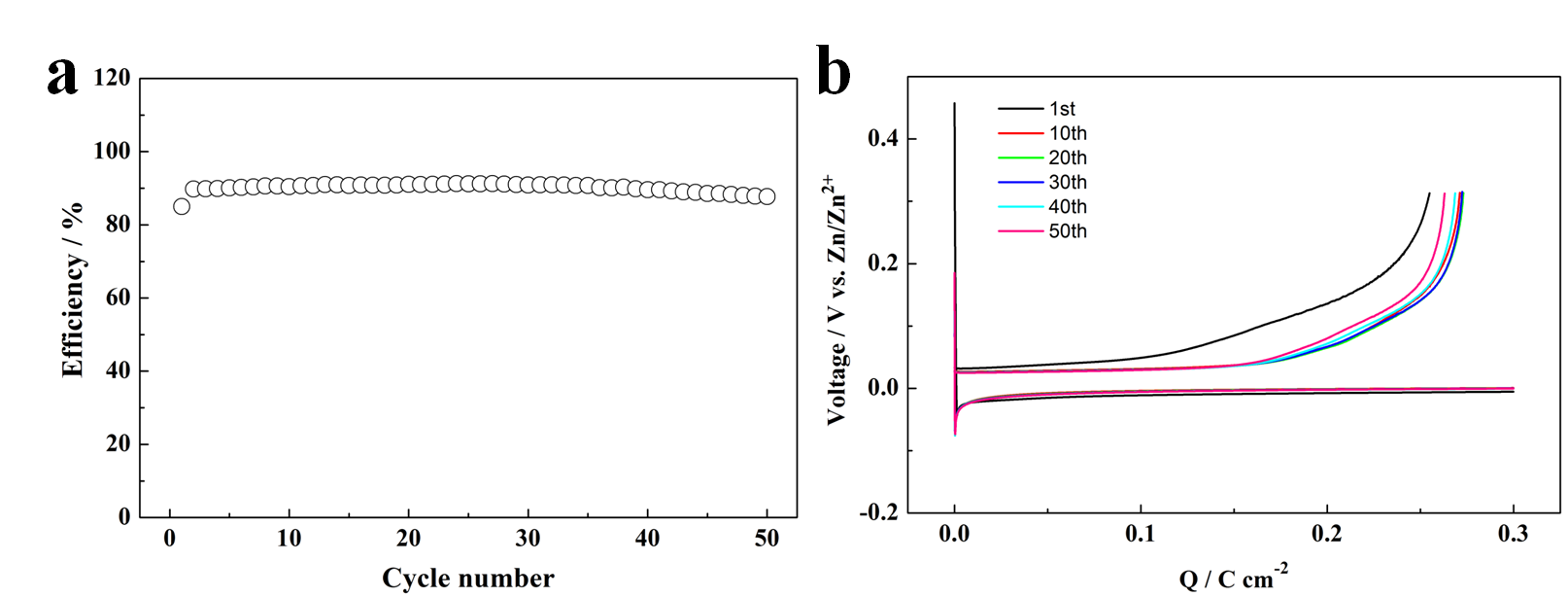


**Fig. S17**. (a) Coulombic efficiency of zinc anode in the 3M ZnSO_4_ electrolyte. The average Coulombic efficiency during 50 cycles is ~90%, due to slight water splitting on zinc anode during charge/discharge processes. (b) Discharge/charge voltage profiles at a current density 0.5 mA cm^-2^ of zinc deposition and stripping in the 3M ZnSO_4_ electrolyte. The relatively slow decay of the capacity of the zinc anode suggests good electrochemical stability.


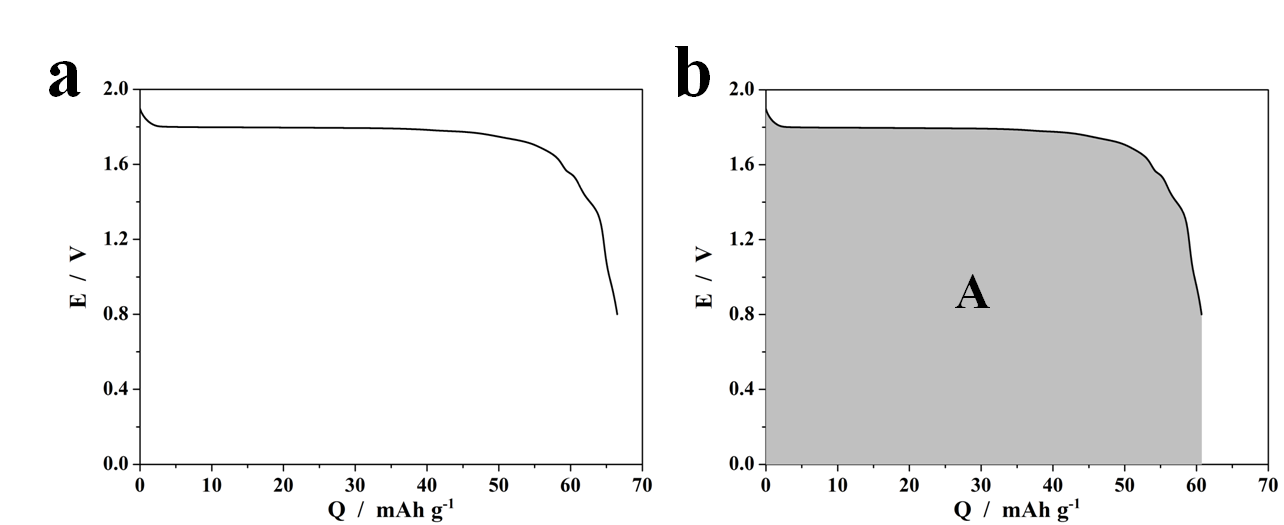


**Fig. S18.** Discharging curves of aqueous zinc-ion battery at 60 mA g^-1^. (a) The specific capacity Q is based on the mass of active cathode materials. (b) The specific capacity Q is based on the total mass of active electrode materials.

The specific energy density which is based on the total mass of active electrode materials, Wh/kg, is the energy that can be derived per total mass of active electrode material. It is the product of the specific capacity based on the total mass of active electrode materials Q (mAh g^-1^) and the operating voltage E (V) in one full discharge cycle. Therefore, the specific energy derived is calculated by integrating E over Q: specific energy density = ∫ E dQ. Consequently, the area of zone A (grey color) in Fig. S12b equals to the specific energy density based on the total mass of active electrode materials. The area of zone A is calculated to be 104.9 Wh kg^-1^. The specific capacity based on the total mass of active electrode materials as shown in Fig. S12b is 60.7 mAh g^-1^. According to the following equation: Energy = Capacity × Voltage, the average potential value is calculated to be 1.73 V (104.9 Wh kg^-1^/60.7 mAh g^-1^).


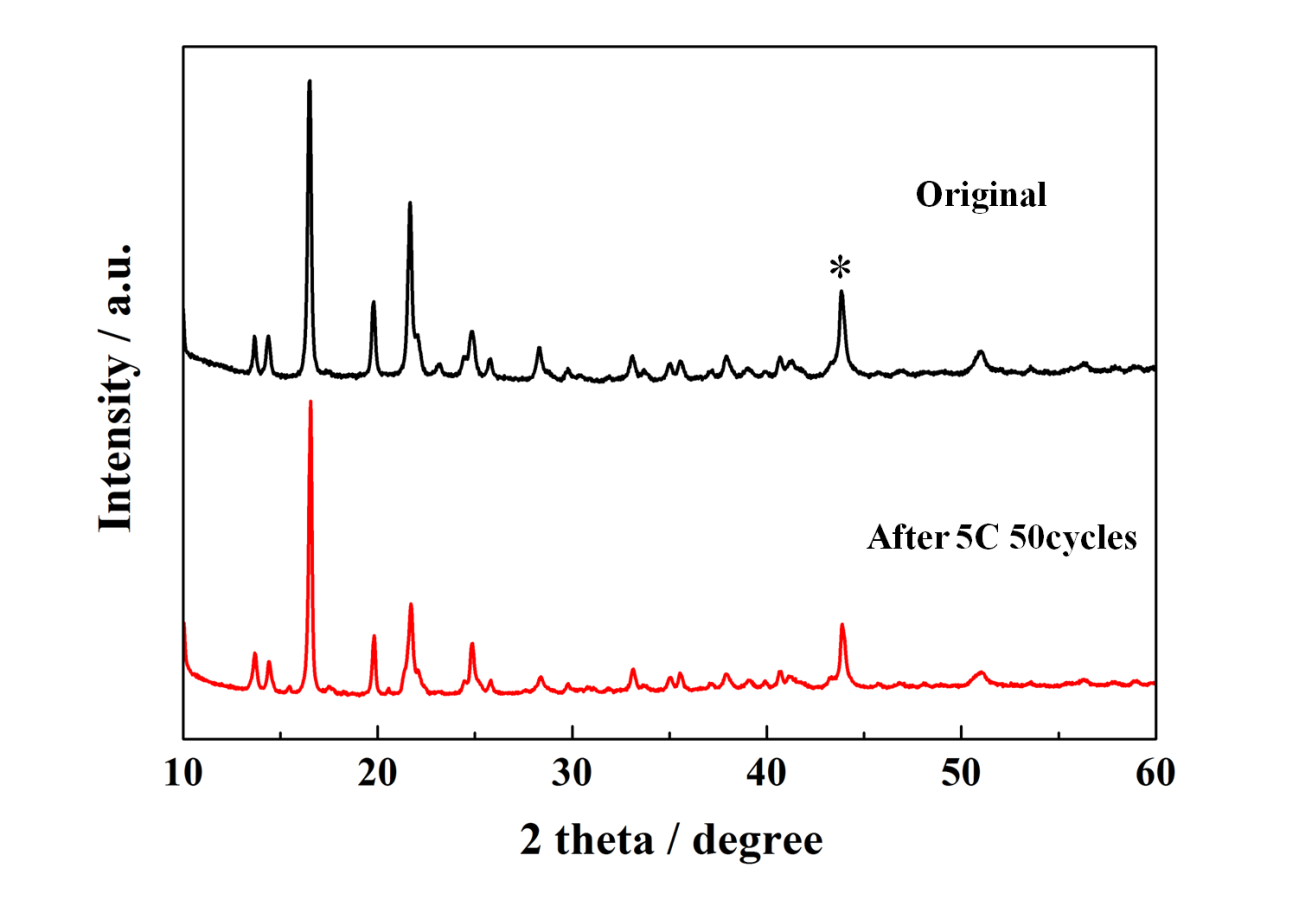


**Fig. S19**. XRD patterns of C-RZnHCF electrode before and after 50 cycles (*, the peak of iron grid).


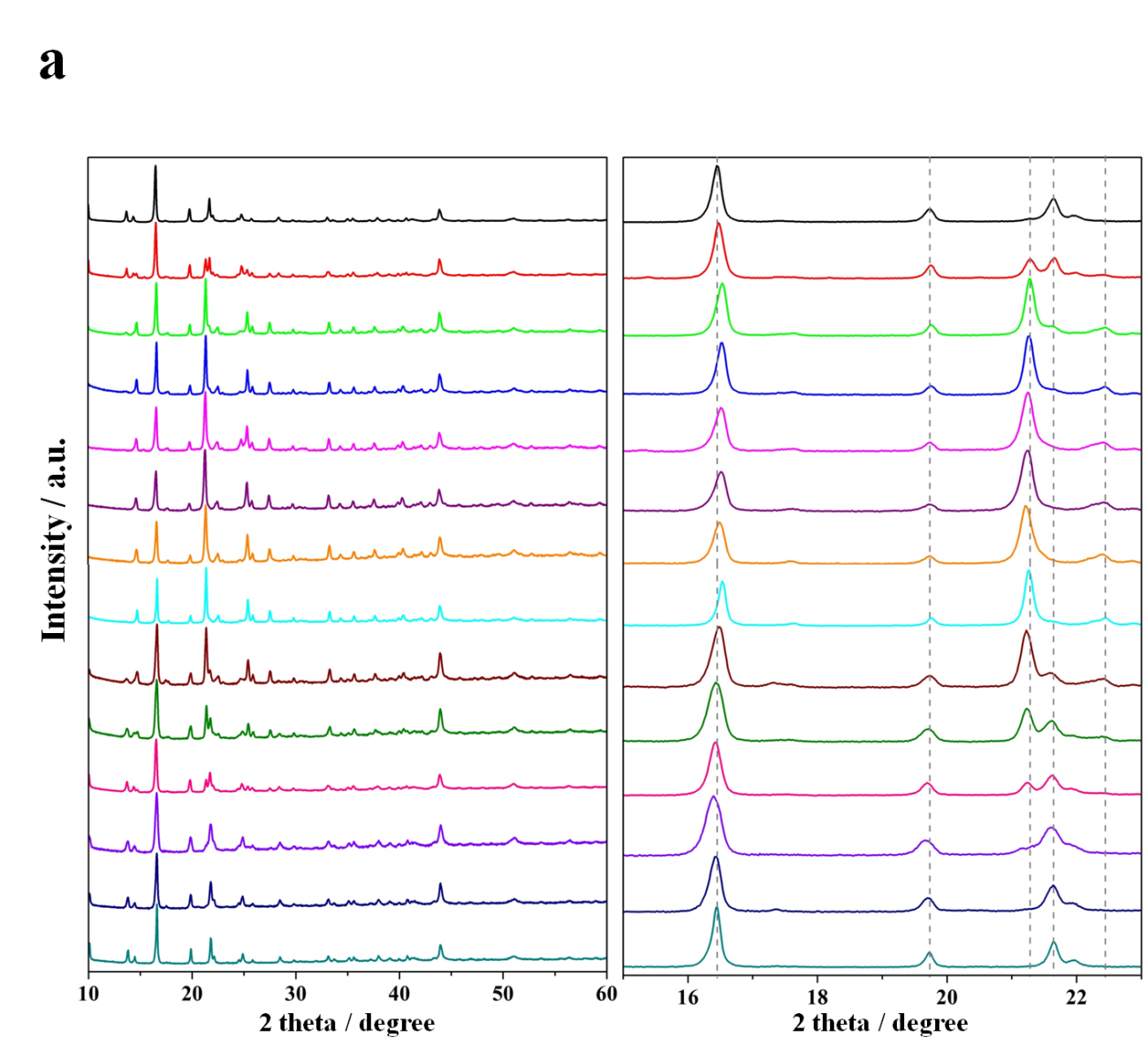


**Fig. S20**. *Ex situ* XRD patterns of C-RZnHCF electrode in 3M ZnSO_4_ electrolytes at various discharge and charge states of the second cycle.


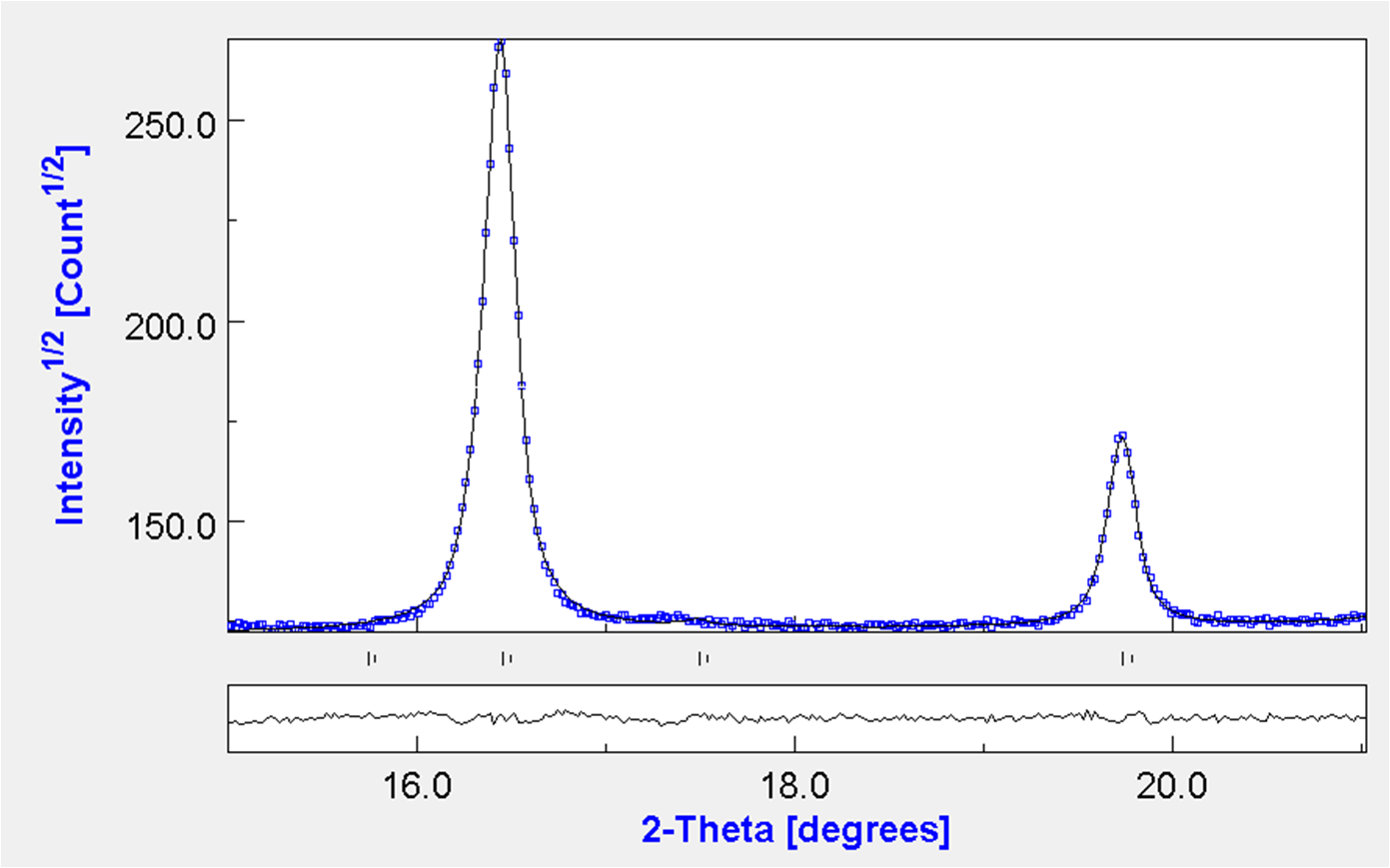


**Fig. S21**. XRD patterns observed (blue cube), calculated (black line), and difference profile (lower pane) for the Rietveld refinement of the pristine C-RZnHCF electrode from 15~21°. sig = 1.62, R_w_ = 1.10 R_wnb_ = 0.85 R_b_ = 0.89 R_exp_ = 0.68, and lattice parameter, a = 12.265 Å.
